# Heterologous Complementation Studies With the YscX and YscY Protein Families Reveals a Specificity for *Yersinia pseudotuberculosis* Type III Secretion

**DOI:** 10.3389/fcimb.2018.00080

**Published:** 2018-03-16

**Authors:** Jyoti M. Gurung, Ayad A. A. Amer, Monika K. Francis, Tiago R. D. Costa, Shiyun Chen, Anton V. Zavialov, Matthew S. Francis

**Affiliations:** ^1^Department of Molecular Biology, Umeå University, Umeå, Sweden; ^2^Umeå Centre for Microbial Research, Umeå University, Umeå, Sweden; ^3^Key Laboratory of Special Pathogens and Biosafety, Wuhan Institute of Virology, Chinese Academy of Sciences Wuhan, Wuhan, China; ^4^Department of Chemistry, University of Turku, Turku, Finland

**Keywords:** T3S chaperone, secretion hierarchy, substrate sorting, LcrH/SycD, YscV, protein–protein interaction

## Abstract

Type III secretion systems harbored by several Gram-negative bacteria are often used to deliver host-modulating effectors into infected eukaryotic cells. About 20 core proteins are needed for assembly of a secretion apparatus. Several of these proteins are genetically and functionally conserved in type III secretion systems of bacteria associated with invertebrate or vertebrate hosts. In the Ysc family of type III secretion systems are two poorly characterized protein families, the YscX family and the YscY family. In the plasmid-encoded Ysc-Yop type III secretion system of human pathogenic *Yersinia* species, YscX is a secreted substrate while YscY is its non-secreted cognate chaperone. Critically, neither an *yscX* nor *yscY* null mutant of *Yersinia* is capable of type III secretion. In this study, we show that the genetic equivalents of these proteins produced as components of other type III secretion systems of *Pseudomonas aeruginosa* (PscX and PscY), *Aeromonas* species (AscX and AscY), *Vibrio* species (VscX and VscY), and *Photorhabdus luminescens* (SctX and SctY) all possess an ability to interact with its native cognate partner and also establish cross-reciprocal binding to non-cognate partners as judged by a yeast two-hybrid assay. Moreover, a yeast three-hybrid assay also revealed that these heterodimeric complexes could maintain an interaction with YscV family members, a core membrane component of all type III secretion systems. Despite maintaining these molecular interactions, only expression of the native *yscX* in the near full-length *yscX* deletion and native *yscY* in the near full-length *yscY* deletion were able to complement for their general substrate secretion defects. Hence, YscX and YscY must have co-evolved to confer an important function specifically critical for *Yersinia* type III secretion.

## Introduction

Type III secretion (T3S) is an effective means for many different Gram-negative bacteria to deliver proteins into diverse eukaryotic cell types. This is a common virulence mechanism of many harmful pathogens (Buttner, 2012; Portaliou et al., 2016), but is also useful for bacteria relying on a symbiotic lifestyle or for survival in the environment (Pallen et al., 2005), and for the biosynthesis of the flagella apparatus (Erhardt et al., 2010). Upon artificial induction, a T3S system (T3SS) can also secrete substrates into laboratory culture media and this requires a complex of ~20 proteins that all together span both bacterial membranes (inner and outer), the peptidoglycan layer and which also protrudes out from the surface. This apparatus has been purified from a few different bacteria, taking on the appearance of a syringe equipped with needle (often termed the “needle complex”) (Kubori et al., 1998; Blocker et al., 1999, 2001; Kimbrough and Miller, 2000; Tamano et al., 2000, 2002; Sekiya et al., 2001; Sukhan et al., 2001; Journet et al., 2003; Marlovits et al., 2004; Mueller et al., 2005). Generally, protein cargo is thought to be secreted through the needle complex (Radics et al., 2014), and these can be divided into three categories—“early” substrates that make up the outer needle, “middle” substrates comprising the pore-forming translocon proteins and “late” host-modulating effector proteins that with assistance from the translocon are targeted to the eukaryotic cell interior (Osborne and Coombes, 2011; Buttner, 2012; Dewoody et al., 2013).

About 10 proteins exist in the needle complex that are common to all known non-flagella and flagella T3SSs (Francis et al., 2004; Buttner, 2012; Portaliou et al., 2016). Moreover, thorough analysis of sequence similarities can classify all T3SSs into evolutionary distinct nodes (Troisfontaines and Cornelis, 2005; Abby and Rocha, 2012). Among the non-flagella T3SSs, a major node is the *inv*/*spa* system encoded by the *Salmonella* pathogenicity island 1 (SPI-1) of *Salmonella enterica* and including systems from *Shigella* sp. (*mxi*/*spa*), *Escherichia coli* (*evi*/*epa*) and *Burkholderia* sp. (*inv*/*spa*). Another major node is characterized by the *ysc* system, encoded on a common virulence plasmid of human pathogenic *Yersinia* sp., and includes systems found in *Pseudomonas aeruginosa* (*psc*), selected *Aeromonas* sp. (*asc*), *Photorhabdus luminescens* (*lsc/sct*) and certain *Vibrio* sp. (*vsc*). Unique to this node are a number of genetically distinct T3SS components not represented in other nodes. Two examples of this are the components YscX and YscY.

The functional role of YscX and YscY in T3S remains enigmatic. In *Yersinia*, a mutant lacking either *yscX* or *yscY* allele poorly synthesizes Yop substrates and their secretion is abolished (Iriarte and Cornelis, 1999; Day and Plano, 2000; Bröms et al., 2005). Moreover, YscX is a T3SS substrate that prior to secretion is stabilized in the cytoplasm by the YscY chaperone (Iriarte and Cornelis, 1999; Day and Plano, 2000). Interestingly, an *in silico* analysis revealed that YscY possesses three tandom tetratricopeptide repeats (Pallen et al., 2003) that are important for function of the translocator class of T3S chaperones (Bröms et al., 2006; Edqvist et al., 2006; Buttner et al., 2008; Lunelli et al., 2009; Job et al., 2010; Singh et al., 2013; Kim et al., 2014). These studies taken together indicate that YscX could be a structural component of the needle complex, while YscY would be necessary to stabilize pre-made pools of YscX and to ensure correct temporal YscX secretion. However, a recent study could find no support for YscX association with the needle, but rather together with YscY was found associated with YscV (alternatively known as LcrD), a core structural inner membrane component of all T3SSs (Diepold et al., 2012).

In a study of *P. aeruginosa* T3S, it was reported that the YscY homolog, Pcr4 (from here on termed PscY for consistency), is actually the secreted component, and not the YscX homolog, Pcr3 (here on termed PscX) (Yang et al., 2007). Despite this anomaly, the YscX/PscX and YscY/PscY components otherwise appear to serve somewhat analogous functions in their respective T3SSs (Bröms et al., 2005; Yang et al., 2007). Moreover, expression of *yscX* and *yscY* in the corresponding *P. aeruginosa* mutants can efficiently restore T3S (Bröms et al., 2005). Yet specific differences must exist since neither *pscX* nor *pscY* alone, or even when expressed in combination, could complement the T3S defects observed in *Yersinia* mutants lacking the corresponding alleles (Bröms et al., 2005). A known difference is the YscY interaction with the LcrH chaperone (also known as SycD) that has an undisclosed role in T3SS regulation in *Yersinia* (Francis et al., 2001; Bröms et al., 2005); the equivalent interaction between PscY and the PcrH chaperone in *P. aeruginosa* has not been detected (Broms et al., 2003; Bröms et al., 2005). Moreover, it is not yet known whether PscX and PscY complex with PscV (the YscV homolog) as do the three equivalent *Yersinia* components (Diepold et al., 2012).

Hence, in this study we further investigated the notion that an YscX-YscY complex has evolved molecular attributes unique to T3SS function in *Yersinia*. We analyzed T3S from *Yersinia yscX* or *yscY* mutants ectopically expressing *in trans* homologous alleles from *Aeromonas salmonicida* and *A. hydrophila* (termed *ascX*_*As*_/*ascX*_Ah_ and *ascY*_*As*_ /*ascY*_Ah_), *P. luminescens* (termed *sctX* or *sctY*), *V. harveyi* and *V. parahaemolyticus* (termed *vscX*_*Vh*_ /*vscX*_Vp_ and *vscY*_*Vh*_ /*vscY*_Vp_) or *P. aeruginosa* (PscX and PscY). No combination resulted in restoration of T3S, despite confirmation of reciprocal interactions giving rise to the equivalent of YscX-YscY bipartite and YscX-YscY-YscV tripartite complexes. This suggested that the YscX-YscY complex serves an exclusive T3SS function in *Yersinia*.

## Materials and methods

### Strains, plasmids, and growth conditions

Bacterial strains and plasmids used in this study are listed in Table S1. Bacteria were routinely cultivated in Lysogeny broth (LB) (Bertani, 2004) or LB agar at either 26°C (*Yersinia pseudotuberculosis*) or 37°C (*E. coli*) with aeration. When required, the antibiotics carbenicillin (Cb), kanamycin (Km) and gentamicin (Gm) were added to laboratory culture media at the final concentrations of 100 μg per ml, 50 μg per ml and 20 μg per ml, respectively. Analysis of T3SS by *Y. pseudotuberculosis* occurred at 37°C in Brain Heart Infusion (BHI) broth. Media containing Ca^2+^ ions was the non-inducing condition (BHI supplemented with 2.5 mM CaCl_2_), while media devoid of Ca^2+^ ions was the inducing condition (BHI supplemented with 20 mM MgCl_2_ and 5 mM Ethylene glycol-bis-(β-aminoethyl ether)-N,N,N',N'-tetraacetic acid). To stimulate promoter activity from the expression vectors pMMB67EHgm and/or pMMB208, isopropyl β-D-1-thiogalactopyranoside (IPTG) at a final concentration of 0.4 mM was added.

The *Saccharomyces cerevisiae* reporter strain AH109 or Y190 was maintained by growth at 30°C in YEP broth [2% (w/v) peptone, 1% (w/v) yeast extract, 2% (v/v) glucose] or agar [YEP broth with 2% (w/v) agar]. pGBKT7 derived plasmids were sustained in yeast by growth on SD synthetic minimal medium (0.67% (w/v) Yeast nitrogen base without amino acids, 300 μgml^−1^ L-isoleucine, 1.5 mgml^−1^ L-valine, 200 μgml^−1^ L-adenine hemisulphate salt, 200 μgml^−1^ L-arginine HCl, 200 μgml^−1^ L-histidine HCl monohydrate, 1 mgml^−1^ L-leucine, 300 μgml^−1^ L-lysine HCl, 200 μgml^−1^ L-methionine, 500 μgml^−1^ L-phenylalanine, 2 mgml^−1^ L-threonine) while pBridge derived plasmids were grown on the same media minus methionine. Yeast containing pGADT7 derivatives were cultured on similar SD media with the exception that leucine was replaced with 200 μgml^−1^ L-tryptophan.

### PCR amplification and sequence analysis

Amplified DNA fragments were obtained by PCR using the appropriate oligonucleotide combinations listed as online Supplementary Information (Table S2). These were synthesized by either DNA Technology A/S (Aarhus, Denmark), TAG Copenhagen A/S (Copenhagen, Denmark) or Sigma-Aldrich Sweden AB (Stockholm, Sweden). Amplified fragments were confirmed to be mutation free by first cloning into pCR®4-TOPO TA (Invitrogen AB, Stockholm, Sweden) or pTZ57R using the InsTAclone PCR cloning kit (Thermo Fisher Scientific, Gothenburg, Sweden) and then via commercial sequencing (Eurofins MWG Operon, Ebersberg, Germany or GATC Biotech AB, Solna, Sweden).

### Phylogenetic analysis of YscX and YscY protein families

Protein sequences corresponding to YscX and YscY protein family were mined from National Center for Biotechnology Information (https://www.ncbi.nlm.nih.gov/sites/entrez) and phylogenetic trees obtained by the Neighbor-Joining method using MEGA6 (Molecular Evolutionary Genetics Analysis) (Tamura et al., 2013). Briefly, proteins were aligned by ClustalW using default settings and the unaligned regions and gaps were trimmed. The significance of the phylogenetic grouping was assessed using confidence level for 1,000 replicates. The trees were rooted with genetically related *Bordetella pertusis* I475 YscX-like or YscY-like protein as the respective out-groups.

### Generation of bacterial protein expression constructs

Constructs containing the *yscX* and *yscY* alleles from *Y. pseudotuberculosis* and *pscX* and *pscY* alleles from *P. aeruginosa* have been described previously (Bröms et al., 2005). *ascX* and *ascY* were amplified from both *A. salmonicida* subsp. *salmonicida* JF2267 (a gift from Joachim Frey, Universität Bern, Switzerland) and *A. hydrophila* AH-3 (Juan Tomás, Universidad de Barcelona, Spain). *vscX* and *vscY* were amplified from both *Vibrio harveyi* BB120 (Debra Milton, Umeå University, Sweden) and *V. parahaemolyticus* RIMD2210633 (Tetsuya Iida, Osaka University, Japan). Finally, *sctX* and *sctY* were amplified from *P. luminescens* TT01 (Rif^R^) (David Clarke, University of Bath, United Kingdom). For the complementation assay in *Y. pseudotuberculosis*, with one exception all alleles were cloned individually or together as a dual expression construct into *Eco*RI-*Bam*HI digested pMMB67EHgm. (The *vscY* allele from *V. harveyi* was digested by *Bam*HI-*Pst*I.) To examine stable protein expression in *Y. pseudotuberculosis*, with two exceptions all PCR amplified alleles with a 5-prime FLAG™ epitope were cloned individually into *Bam*HI-*Eco*RI digested pMMB208. (The *yscY* allele from *Y. pseudotuberculosis* and the *vscY* allele from *V. harveyi* was digested by *Pst*I-*Bam*HI.) The same approach was utilized to generate expression constructs of codon-optimized gene variants.

### Construction of yeast plasmids

To investigate reciprocal binding between the products of *yscY, yscX* and related alleles, a series of constructs were generated in the yeast two-hybrid vectors pGBKT7 and pGADT7 (Clontech Laboratories, Palo Alto, CA). Constructs expressing the *yscY* and *pscY* alleles fused to the GAL4 DNA binding domain in pGBKT7 are already described (Francis et al., 2001; Bröms et al., 2005). PCR amplified *ascY, vscY*, and *sctY* alleles were fused to the GAL4 DNA binding domain by cloning into *Eco*RI-*Bam*HI digested pGBKT7. Plasmids expressing the *yscX* and *pscX* alleles fused to the GAL4 activation domain in pGADT7 are already established (Bröms et al., 2005). PCR amplified *ascX, vscX*, and *sctX* alleles were fused to the GAL4 activation domain by cloning into *Eco*RI-*Bam*HI digested pGADT7.

Reciprocal interaction between YscX, YscY, YscV, and related family members were assessed with a yeast three hybrid approach by utilizing a three hybrid GAL4 DNA binding domain pBridge vector (Clontech Laboratories, Palo Alto, CA) and Gal4 activation domain pGADT7 vector. Constructs expressing *yscY* and related alleles were expressed as Gal4 DNA binding fusion protein by introducing into *Eco*RI-*Pst*I digested pBridge downstream of *ADH1* promoter. Additionally, the coding sequences of *yscX* and related alleles were cloned into *Not*I-*Bgl*II sites of pBridge downstream of *MET25* promoter to finally yield pBridge-*yscY*-*yscX* and its variants. Likewise, *yscV* and related members were expressed as GAL4 activation fusion protein from pGADT7. While PCR-amplified *yscV, ascV* and *sctV* alleles were cloned into *Eco*RI-*Xho*I sites, PCR-amplified *pscV* and *vscV* were inserted into *Nde*I-*Bam*HI sites of pGADT7.

### Yeast transformation and the n-hybrid assays

For yeast two-hybrid assay, transformation of the *S. cerevisiae* reporter strain AH109 was performed as described earlier (Francis et al., 2000; Bröms et al., 2005; Amer et al., 2016). Protein interactions from multiple independent transformations were determined by measuring the activation of the *ADE2* reporter gene activation and the *HIS3* reporter gene during growth on tryptophan and leucine minus SD synthetic minimal medium also lacking either adenine or histidine, respectively. The latter also required the addition of 4 mM 3-aminotriazole in the growth media to overcome any risk of false positives (James et al., 1996). Analysis of protein stability in yeast was performed as previously described (Francis et al., 2000).

The yeast three-hybrid assay was performed in *S. cerevisiae* reporter strain Y190 as described previously (Carlo et al., 2007; Glass et al., 2015). Briefly, yeast were co-transformed with pBridge and pGADT7-based vectors and a master plate established by initial growth at 30°C on SD synthetic minimal medium lacking leucine, tryptophan and methionine. The extent of protein interactions were then measured via the activation of the *HIS3* reporter gene upon growth on the above mentioned medium also lacking histidine, but supplemented with 40 mM 3-aminotriazole (3-AT) to neutralize the inherent leakiness of the reporter. Equal amount of colony from each of the transformants were resuspended in water and subjected to 5-fold serial dilutions. An aliquot of each serial dilution was then transferred onto two sets of plates: (1) master plate (SD -Leu, -Trp, -Met) and (2) selective plate (SD −Leu, −Trp, −Met, −His + 40 mM 3-AT). The growth of yeast on the selective plate indicated a positive interaction. As a control, 1 mM methionine was used for near complete repression of *MET25* repressible promoter (Her et al., 2003).

### Analysis of gene transcription by qualitative RT-PCR

Total RNA was isolated from T3SS-induced *Yersinia* cultures as previously described (Carlsson et al., 2007a,b). Briefly, overnight cultures of *Yersinia* grown in a secretion permissive condition (BHI minus Ca^2+^) were diluted into 5 ml fresh media to an optical density (OD) at 600 nm of 0.1. After incubating at 26°C for 30 min, cultures were shifted to 37°C and grown for approximately 90 min to an OD at 600 nm of 0.4 to 0.8. RNA was immediately stabilized by mixing two volumes of RNA protect bacterial reagent (QIAGEN GmbH, Hilden, Germany) with one volume of the bacterial culture. Total RNA was isolated using the NucleoSpin RNA II method (Macherey Nagel, Düren, Germany) that included an on-column DNase treatment. For reverse transcription, 0.2 μg of total RNA was used to generate cDNA by RevertAid H Minus Reverse Transcriptase (RT) system (Thermo Scientific, Vilnius, Lithuania). RNA samples that were not treated with RT were used as negative controls to confirm the absence of contaminating DNA. PCR to confirm the presence of specific transcript was performed using gene-specific primers as listed in Table S2 and the cDNA generated above as template (Carlsson et al., 2007a,b).

Qualitative RT-PCR to determine the stability of mRNA transcript was performed as described elsewhere (Okan et al., 2006; Jeters et al., 2009; Chen and Anderson, 2011) with some modifications. Briefly, *Yersinia* cultures were grown in BHI media lacking Ca^2+^ at 37°C to an OD at 600 nm of 0.4 to 0.8. At time point zero, one volume of bacterial culture was removed and rifampicin to a final concentration of 100 μg/ml was added to the bacterial culture to inhibit *de novo* RNA synthesis. Thereafter, one volume of bacterial cultures was taken at 5, 10, and 20 min time points. RNA was always stabilized by the addition of two volumes of RNA protect bacterial reagent (QIAGEN GmbH, Hilden, Germany). Isolation of total RNA, synthesis of cDNA and qualitative presence of transcript by PCR was performed as described above.

### Synthesis and secretion of type III-secreted substrates

Induction of type III substrate synthesis and secretion from *Y. pseudotuberculosis* was performed as previously described (Francis et al., 2000, 2001). All protein samples were normalized to the amount of bacterial cells at an optical density of 600 nm. Protein associated with whole bacteria was assessed by sampling from pelleted bacterial cultures. Total fraction contained proteins associated within intact bacteria and secreted to the culture medium. Sampling of the cell-free supernatant assessed the secreted protein levels. All protein fractions were separated by SDS-PAGE and subjected to immunoblotting. Detection of *Yersinia* substrates used rabbit polyclonal antisera raised against secreted YopE, YopD, and YopB (AgriSera AB, Vännäs, Sweden).

### Quantification of protein production in *Y. pseudotuberculosis*

Relative protein levels were quantified from protein bands on scanned western blot X-ray films using the gel analysis tool in ImageJ (Schneider et al., 2012). In every case, the lane profile plot area of each protein band of interest was normalized to the corresponding protein band appearing in the same lane in the loading control blot.

## Results

### Amino acid identity among the YscX and YscY protein families

*In silico* analysis has identified up to 9 core structural components in all T3SSs (Francis et al., 2004). Additional common components restricted to genetically related T3SS sub-families can also be found. A genetically related T3SSs exists in the notable human and animal pathogens *Y. pestis, Yersinia enterocolitica, Y. pseudotuberculosis*, and *P. aeruginosa*, the marine pathogens *Aeromonas hydrophilia, A. salmonicida, V. harveyi*, and *V. parahaemolyticus*, and the insect pathogen *P. luminescens*. Components common only to these systems include the YscX and YscY protein families. To ascertain genetic relatedness between representatives of the YscX and YscY protein families, amino acid sequences were retrieved from the sequenced genomes at http://www.ncbi.nlm.nih.gov/sites/entrez of the above mentioned bacteria and then aligned with ClustalW (http://www.ebi.ac.uk/Tools/clustalw/index.html). The evolutionary pattern was then examined by generating a phylogenetic tree rooted against the genetically related YscX-like and/or YscY-like protein from *B. pertusis* I475 by using the neighbor-joining method (Tamura et al., 2013; Bhattacharyya et al., 2017).

**Figure 1:**
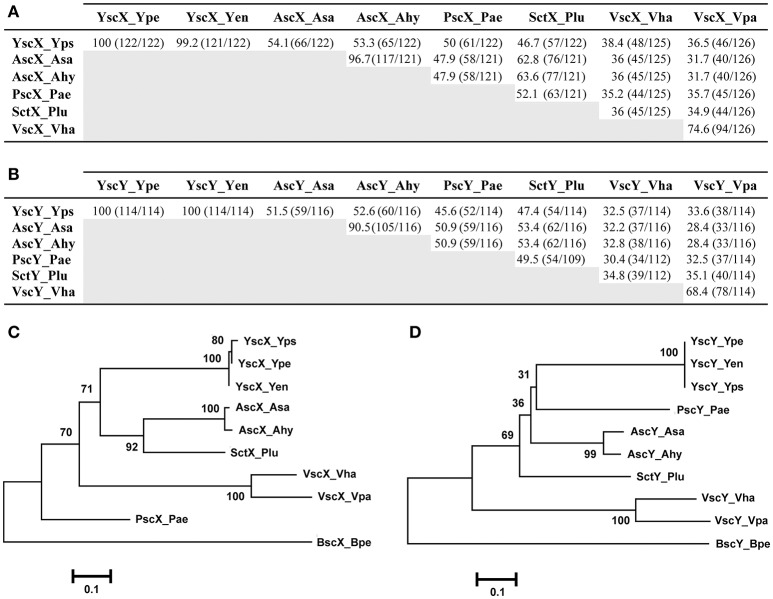
Amino acid sequence relatedness between the YscX and YscY protein families. Amino acid sequence identity was determined by BLASTP analysis (BLASTP 2.8.0+) (Altschul et al., 1997, 2005) for the YscX protein **(A)** and YscY protein **(B)** families. Numbers outside of parenthesis indicate percent amino acid identity. This is calculated from a ratio of the numbers inside parentheses; the left number indicating the number of identical residues, and the right number indication the total number of overlapping residues in a pairwise alignment. Representative sequences were retrieved from the NCBI genome database archived with the following GI reference numbers shown in parentheses: Yps, *Yersinia pseudotuberculosis* (YscX, 51593904 and YscY, 51593903); Ype, *Yersinia pestis* (YscX, 16082724 and YscY, 16082723); Yen, *Yersinia enterocolitica* (YscX, 4324343 and YscY, 4324342); Asa, *Aeromonas salmonicida* (AscX, 66947966 and AscY, 66947967); Ahy, *Aeromonas hydrophilia* (AscX, 46398260 and AscY, 46398261); Pae, *Pseudomonas aeruginosa* (PscX, 62865832 and PscY, 62865833); Plu, *Photorhabdus luminescens* (SctX, 36787060 and SctY, 36787059); Vha, *Vibrio harveyi* (VscX, 41834176 and VscY, 41834175); Vha, *Vibrio parahaemolyticus* (VscX, 28898438 and VscY, 28898437). Alignments of these sequences were used to generate phylogenetic trees depicting relationships between amino acid sequences of YscX-like **(C)** and YscY-like **(D)** family members. The neighbor-joining phylogenetic tree was generated using full length amino acid sequences of YscX and YscY homologs. Genetically related YscX-like and YscY-like protein sequences from *Bordetella pertusis I475* were used as an outgroup. Values next to each branch node represent bootstrap percentages for 1,000 replicates. The scale bar represents the number of amino acid substitutions per site.

Based on amino acid sequence, the percent amino acid identity to YscX (122 aa) ranged from 54.1% over a 122 residue overlap (*A. salmonicida*) to 36.5% across a 126 residue overlap (*V. parahaemolyticus*) (Figure 1A). Apart from the near complete identity of YscX among the three *Yersinia* species, most relatedness occurred between the AscX proteins produced by the two *Aeromonas* species (~97% identical) and the VscX proteins produced by the two *Vibrio* species (~75%) (Figure 1A). Consistent with this, a phylogenetic tree analysis revealed that they each formed a separate clade distinct from other homologs (Figure 1C).

Similarly, amino acid sequence identity to YscY (114 aa) from *Yersinia* ranged between 52.6% (*A. hydrophila*) over a 116 residue overlap down to 32.5% (*V. harveyi*) over a 114 residue overlap (Figure 1B). Apart from the YscY proteins, the AscY proteins displayed most similarity to each other (~91% identical), followed by the VscY proteins (~68%), consistent with them forming separate phylogenetic clades (Figure 1D). In addition, the *P. luminescens* proteins SctX and SctY were most similar to the AscX and AscY counterparts from *Aeromonas* species (~63 and ~61%, respectively) (Figures 1A,B) and they all grouped close together on the phylogenetic tree (Figures 1C,D). Finally, among the three human pathogenic *Yersinia* species, YscY amino acid sequence was identical, whereas in YscX the amino acid glutamine at position 29 in both *Y. pseudotuberculosis* and *Y. pestis* existed as an aspartate residue in *Y. enterocolitica* (Figure S1). This subtle sequence divergence of YscX from *Y. enterocolitica* is reflected in the phylogenetic tree (Figure 1C).

In summary, this analysis reveals that YscX and YscY generally share most genetic relatedness with equivalent proteins from the two *Aeromonas* sp. Moreover, the YscX and YscY homologs that have diverged the most stem from the two *Vibrio* sp. This is in concordance with the phylogeny of non-flagella-T3SS of other core Ysc structural components (Troisfontaines and Cornelis, 2005; Romano et al., 2016).

### Reciprocal chaperone-substrate interactions

**Figure 2:**
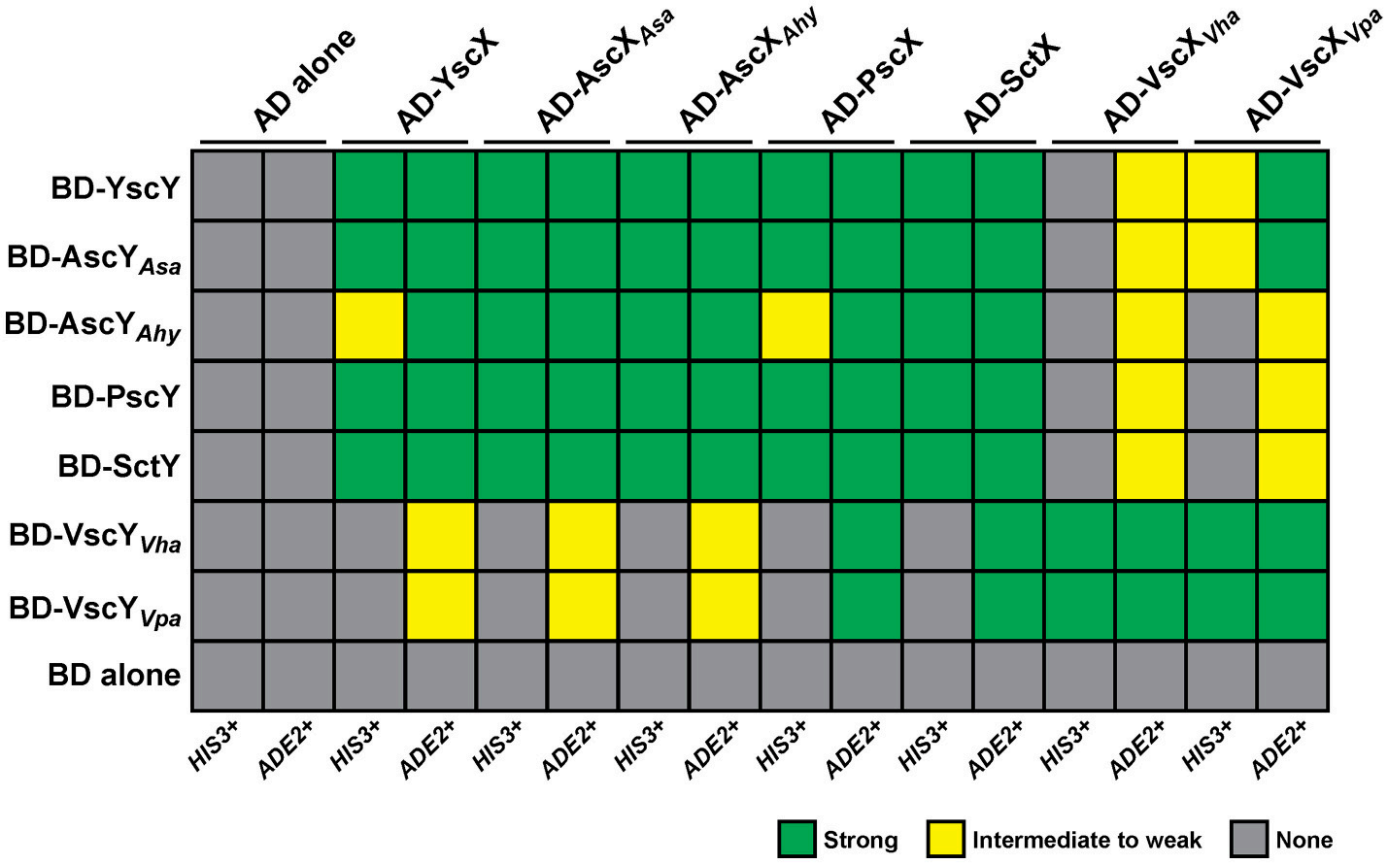
Summary of the reciprocal binding between members of the YscX and YscY protein families. Protein–protein interactions were determined using the yeast two-hybrid assay. YscX family members were fused to the *GAL4* activation domain (in pGADT7), whereas YscY family members were fused to the *GAL4* DNA binding domain (in pGBKT7). Pairwise transformations were performed in *S. cerevisiae* AH109 (Clontech Laboratories) that contained the *HIS3* and *ADE2* reporter genes. Strength of interactions were determined by the extent of growth on minimal medium devoid of histidine or adenine and recorded after day 4. Green shade indicates robust yeast growth (strong binary interaction), yellow shade reflects modest growth (moderate interaction) and gray shade specifies no growth (no interaction). Due to an intrinsic leakiness with the *HIS3* reporter, 4 mM 3-aminotriazole was added to histidine dropout media to suppress false positives (James et al., 1996).

A cornerstone of YscX and YscY function appears to be their ability to interact with each other (Day and Plano, 2000; Bröms et al., 2005), and this seems true also of PscX and PscY function in *P. aeruginosa* T3S (Bröms et al., 2005; Yang et al., 2007). The yeast two-hybrid system has proven to be a reliable tool to demonstrate reciprocal interactions of YscX/PscX with YscY/PscY (Francis et al., 2001; Bröms et al., 2005). Thus, we utilized this approach to examine the interaction reciprocity between the representative YscX and YscY family members detailed in Figure 1. The PCR amplified *yscX*-related alleles were all separately cloned into the pGADT7 vector to establish fusions to the C-terminus of the *GAL4* activation domain (AD). Additionally, the PCR amplified *yscY*-related alleles were all individually cloned into the pGBKT7 vector to establish fusions to the C-terminus of the *GAL4* DNA binding domain (BD). The various pair-wise combinations of vectors expressing a AD and BD fusion were established in *S. cerevisiae* AH109 containing an *ADE2* and *HIS3* reporter gene. Interactions between YscX-like and YscY-like proteins were determined by the ability of transformed yeast to grow on either minimal media lacking histidine (but supplemented with 3-aminotriazole at a final concentration of 4 mM) or adenine. Interaction specificity was confirmed by the failure of a particular strain to grow on this same media after being cured of either the AD or BD expressing plasmid (data not shown). Reciprocal chaperone (YscY-like)-substrate (YscX-like) interactions could be observed for every possible combination (Figure 2, Figure S2). However, YscY-like variants derived from *Y. pseudotuberculosis, P. aeruginosa, Aeromonas* species, and *P. luminescens* all interacted better with YscX-like proteins also derived from these same bacteria. In contrast, these proteins consistently displayed weaker interactions with their more distantly related counterparts originating from *Vibrio* species (Figure 2, Figure S2). Importantly however, this was not a reflection of reduced protein expression, since cognate proteins derived from *V. harveyi* and *V. parahaemolyticus* demonstrated strong reciprocal binding with each other (Figure 2, Figure S2). Hence, reciprocal interactions between YscY- and YscX-like proteins are all generated in a yeast two-hybrid system. Conservation of these interactions is suggestive of a common functional requirement in T3S.

### Reciprocal ternary interactions between YscX, YscY and the inner membrane component YscV indicate formation of a conserved substrate secretion sorting complex

**Figure 3:**
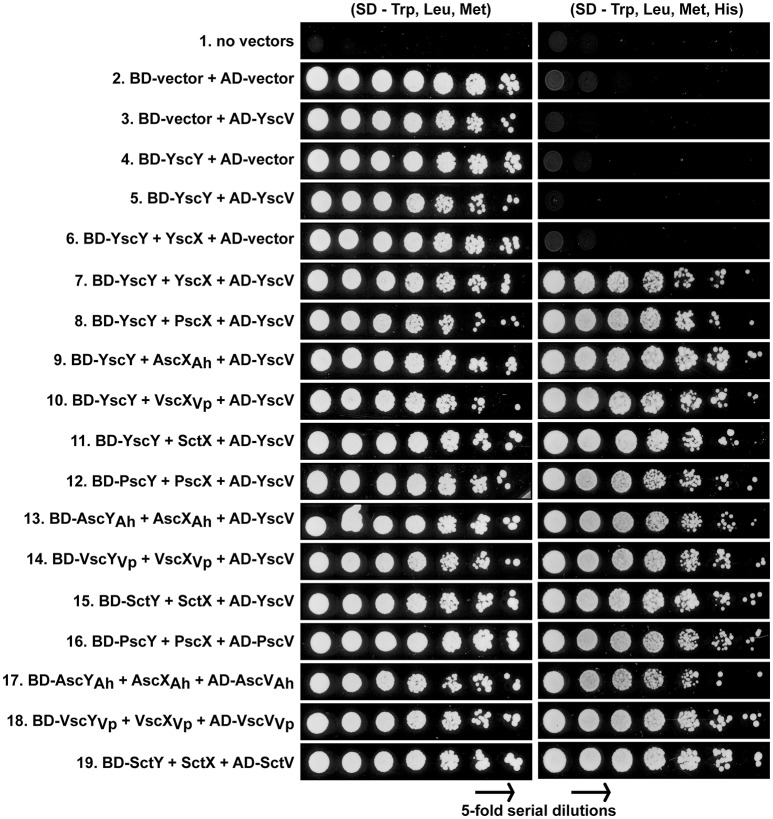
Reciprocal interactions between members of YscX, YscY, and YscV protein families. Different protein members of the YscY family were produced as recombinant GAL4 binding domain fusions from the pBridge vector. From this vector were also produced native forms of YscX protein family members to act as bridging proteins. Different protein members of the YscV family were produced as recombinant GAL4 activation domain fusions from the pGADT7 vector. As indicated, pairwise combinations of pBridge and pGADT7 derivatives were co-transformed into the yeast strain *S. cerevesiae* Y190. Five-fold serial dilutions of transformed yeast were grown on a synthetic dropout (SD) agar plates lacking tryptophan (Trp) and leucine (Leu) for maintenance of the plasmid pairs and methionine (Met) to induce production of YscX-like proteins. The strength of interaction was studied by growing yeast in equivalent media lacking Histidine (His). A concentration of 40 mM 3-aminotriazole was added to reduce leaky *HIS3* expression. The result shown is a representative of three independent experiments.

Together YscX and YscY co-interact with YscV, a highly conserved T3SS component that makes up part of the inner membrane export apparatus (Diepold et al., 2012). This YscX-YscY-YscV tripartite complex helps to facilitate the temporal export of early T3S substrates (Diepold et al., 2012). We utilized the yeast three hybrid approach to verify that YscX and YscY together form a ternary complex with YscV (Figure 3). Y190 yeast competent cells were co-transformed with pBridge vector carrying P_ADH1_-BD-YscY/p_MET25_-YscX and pGADT7 carrying p_ADH1_-AD-YscV. Transformed yeast cells were grown on SD minimal medium lacking histidine (but supplemented with 40 mM 3-aminotriazole) either in the absence or presence of methionine as a means to control output from the leaky *MET25* promoter (Carlo et al., 2007). Expression of *yscX* under the control of *MET25* promoter can be repressed in the presence of 1 mM methionine and activated in methionine-free media. Based on the extent of yeast growth in the absence of methionine, we could verify the formation of a YscY-YscX-YscV complex (Figure 3, panel 7). Importantly, the interaction was comparatively diminished upon growing yeast transformants on media containing 1 mM methionine. As expected, YscX did not bind to YscV in the absence of YscY (unpublished data), nor did YscY bind to YscV in the absence of YscX (Figure 3, panel 5). Moreover, transformation of empty vectors, YscV or YscX-YscY alone did not permit auto-activation of *HIS3* reporter gene. These data demonstrated the suitability of the yeast three hybrid system for detecting the YscY-YscX-YscV interaction. Hence, we then tested if YscY-like, YscX-like, and YscV-like proteins from *P. aeruginosa, A. hydrophila, V. parahaemolyticus*, and *P. luminescens* have evolved to maintain the tripartite interaction with their specific counterparts. A significant amount of interaction between the cognate partners was evident in all YscY, YscX, YscV homologs tested (Figure 3, panel 16–19). Given the ability of YscY, YscX, and YscV homologs to maintain tripartite interactions, we then tested the possibility of reciprocal interactions involving the different combinations of family members. A reciprocal interaction between the members of ternary complex was observed in all combinations (Figure 3, panel 8–15). Thus, conservation of this interaction suggests that YscY-like, YscX-like, and YscV-like proteins have coevolved to perform a common T3S function, which is to facilitate the temporal export of early T3S substrates (Diepold et al., 2012).

### Production of YscX and YscY is a strict requirement for T3SS assembly and function in *Y. pseudotuberculosis*

**Figure 4:**
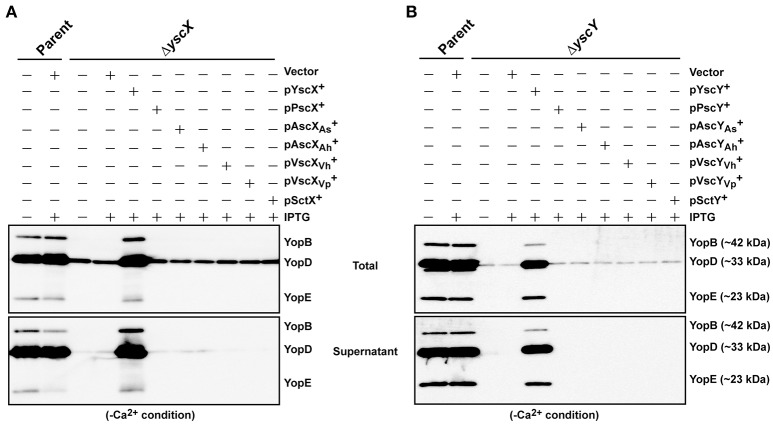
Assessing complementation of YscX and/or YscY function in *Yersinia* type III secretion. Bacteria were grown in BHI medium under secretion-permissive conditions (absence of Ca^2+^). Proteins contained within intact bacteria and secreted to the culture medium (Total) or secreted free to the extracellular medium (Supernatant) were fractionated on a 12% SDS-PAGE and analyzed by immunoblotting using polyclonal rabbit anti-YopB, anti-YopD and anti-YopE antiserum. IPTG was added to a final concentration of 0.4 mM where indicated. **(A)** Parent *Y. pseudotuberculosis* and Δ*yscX* complemented with pMMB67EHgm or pMMB67EHgm-encoded YscX family members. Strains: Parent (YPIII/pIB102); Complemented YPIII/pIB102, pMMB67EHgm (Vector);Δ*yscX* (YPIII/pIB880); complemented YPIII/pIB880, pMMB67EHgm (Vector); complemented YPIII/pIB880, pJEB291 (YscX^+^); complemented YPIII/pIB880, pJEB295 (PscX^+^); complemented YPIII/pIB880, pMF720 (${\text{AscX}}_{\text{As}}^{+}$); complemented YPIII/pIB880, pMF722 (${\text{AscX}}_{\text{Ah}}^{+}$); complemented YPIII/pIB880, pMF724 (${\text{VscX}}_{\text{Vh}}^{+}$); complemented YPIII/pIB880, pMF725 (${\text{VscX}}_{\text{Vp}}^{+}$); complemented YPIII/pIB880, pMF727 (SctX^+^). **(B)** Parent *Y. pseudotuberculosis* and Δ*yscY* complemented with pMMB67EHgm or pMMB67EHgm-encoded YscY family members. Strains: Parent (YPIII/pIB102); Δ*yscY* (YPIII/pIB890); complemented YPIII/pIB890, pMMB67EHgm (Vector); complemented YPIII/pIB890, pJEB292 (YscY^+^); complemented YPIII/pIB890, pJEB296 (PscY^+^); complemented YPIII/pIB890, pMF721 (${\text{AscY}}_{\text{As}}^{+}$); complemented YPIII/pIB890, pMF723 (${\text{AscY}}_{\text{Ah}}^{+}$); complemented YPIII/pIB890, pMF796 (${\text{VscY}}_{\text{Vh}}^{+}$); complemented YPIII/pIB890, pMF726 (${\text{VscY}}_{\text{Vp}}^{+}$); complemented YPIII/pIB890, pMF728 (SctY^+^). Molecular mass values shown in parentheses were deduced from primary amino acid sequences.

**Figure 5:**
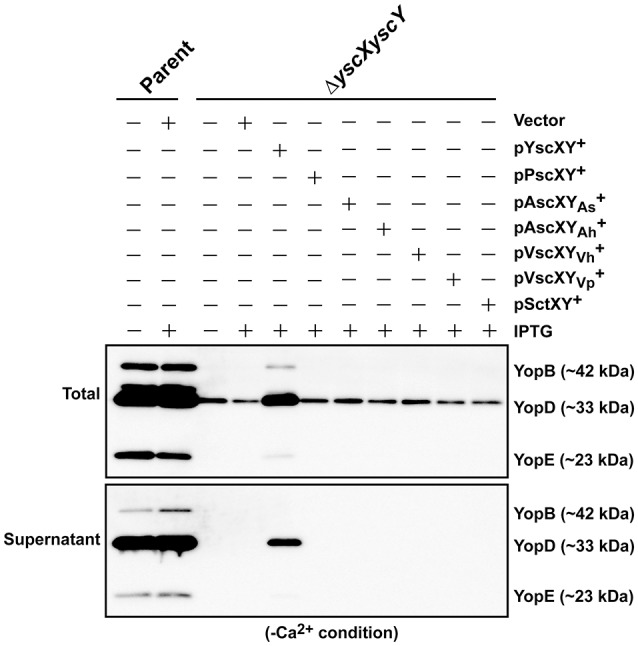
Assessing functional complementation of YscX and YscY by dual expression of substrate and chaperone homologs. Bacteria were grown in BHI medium under secretion-permissive conditions (absence of Ca^2+^). Proteins contained within intact bacteria and secreted to the culture medium (Total) or secreted free into the extracellular medium (Supernatant) were fractionated on a 12% SDS-PAGE and analyzed by immunoblotting using polyclonal rabbit anti-YopB, anti-YopD, and anti-YopE antiserum. IPTG was added to a final concentration of 0.4 mM where indicated. Strains: Parent (YPIII/pIB102); Δ*yscXyscY* (YPIII/pIB881); complemented YPIII/pIB881, pMMB67EHgm (Vector);complemented YPIII/pIB881, pJEB340 (YscX, YscY^+^); complemented YPIII/pIB881, pJEB335 (PscX, PscY^+^); complemented YPIII/pIB881, pMF733 (AscX_As_, ${\text{AscY}}_{\text{As}}^{+}$); complemented YPIII/pIB881, pMF734 (AscX_Ah_, ${\text{AscY}}_{\text{Ah}}^{+}$); complemented YPIII/pIB881, pMF797 (VscX_Vh_, ${\text{VscY}}_{\text{Vh}}^{+}$); complemented YPIII/pIB881, pMF735 (VscX_Vp_, ${\text{VscY}}_{\text{Vp}}^{+}$); complemented YPIII/pIB881, pMF736 (SctX, SctY^+^). Molecular mass values shown in parentheses were deduced from primary amino acid sequences.

Next we asked whether members of the YscX and YscY protein families could restore T3S function to a respective Δ*yscX* or Δ*yscY* null mutant of *Y. pseudotuberculosis*. To achieve this, the various PCR-amplified *yscX*- and *yscY*-like alleles were cloned either separately or together under the control of an IPTG inducible promoter in the expression vector pMMB67EHgm and then conjugated into the appropriate *Y. pseudotuberculosis* background. Following standardization against bacterial cell number, the level of T3S substrates associated with the total bacteria (a mixture of proteins contained within intact bacteria and secreted to the culture supernatant) and secreted free into the culture media was examined from bacteria grown in both T3S restrictive (BHI plus Ca^2+^) and permissive (BHI minus Ca^2+^) conditions. Expectantly, YscX when expressed in a Δ*yscX* null mutant (Figure 4A) and YscY when expressed in a Δ*yscY* null mutant (Figure 4B) restored functional T3S in *Y. pseudotuberculosis*. Moreover, YscX derived from *Y. enterocolitica* could readily complement the T3S defect in the Δ*yscX* null mutant (data not shown). Furthermore, dual expression of both YscX and YscY together in a Δ*yscX, yscY* double mutant also restored functional T3S (Figure 5). On the other hand, no expressed YscX-like or YscY-like protein could restore functional T3S to a Δ*yscX* null mutant (Figure 4A) or to a Δ*yscY* null mutant (Figure 4B), respectively. Additionally, co-expression of the cognate substrate-chaperone pair did not help to restore T3S to a Δ*yscX, yscY* double mutant (Figure 5). These data are consistent with an earlier study which concluded that PscX and PscY from *P. aeruginosa* could not complement the Yop synthesis and secretion defect apparent in a *Y. pseudotuberculosis* mutant lacking the *yscX* and *yscY* alleles respectively (Bröms et al., 2005). Taken all together, these data suggest that YscX-YscY interplay has functional consequences unique to T3S by *Yersinia*.

### Codon optimization fails to improve the complementation efficiency of non-endogenous *yscX*- and *yscY*-like alleles

Complementation may have failed because the non-cognate alleles were not sufficiently expressed. Due to the absence of available antibodies, production of YscX-like and YscY-like proteins in *Y. pseudotuberculosis* was assessed by appending an N-terminal FLAG™ epitope, followed by immunoblotting using an anti-FLAG™ monoclonal antibody. Accumulated levels of recombinant PscX, VscX_Vp_, SctX and to a much lesser extent VscX_Vh_ could be detected (Figure S3A). Additionally, accumulated levels of recombinant PscY, VscX_Vh_, VscX_Vp_, and to a lesser extent SctY could be detected (Figure S3B). However, accumulated levels of all four recombinant Asc proteins were undetectable under these assay conditions (Figure S3). A number of reasons could contribute to the wide disparity in heterologous protein production in *Yersinia*, although it can often be attributed to species-specific GC content and species-specific codon usage (Gustafsson et al., 2004; Yu et al., 2015; Zhou et al., 2016). Indeed, the percent GC content of the individual genomes targeted here do vary considerably—*Y. pseudotuberculosis* (47.6% GC content), *Aeromonas* sp. (61.6%), *P. aeruginosa* (66.6%), *Vibrio* sp. (45.4%), and *P. luminescens* (42.8%) (http://archaea.ucsc.edu/) (Schneider et al., 2006; Chan et al., 2012), and this variance can be assumed to tailor the codon usage within each individual allele that may be incompatible with optimal translation in *Y. pseudotuberculosis* (Gustafsson et al., 2004; Yu et al., 2015; Zhou et al., 2016).

**Figure 6:**
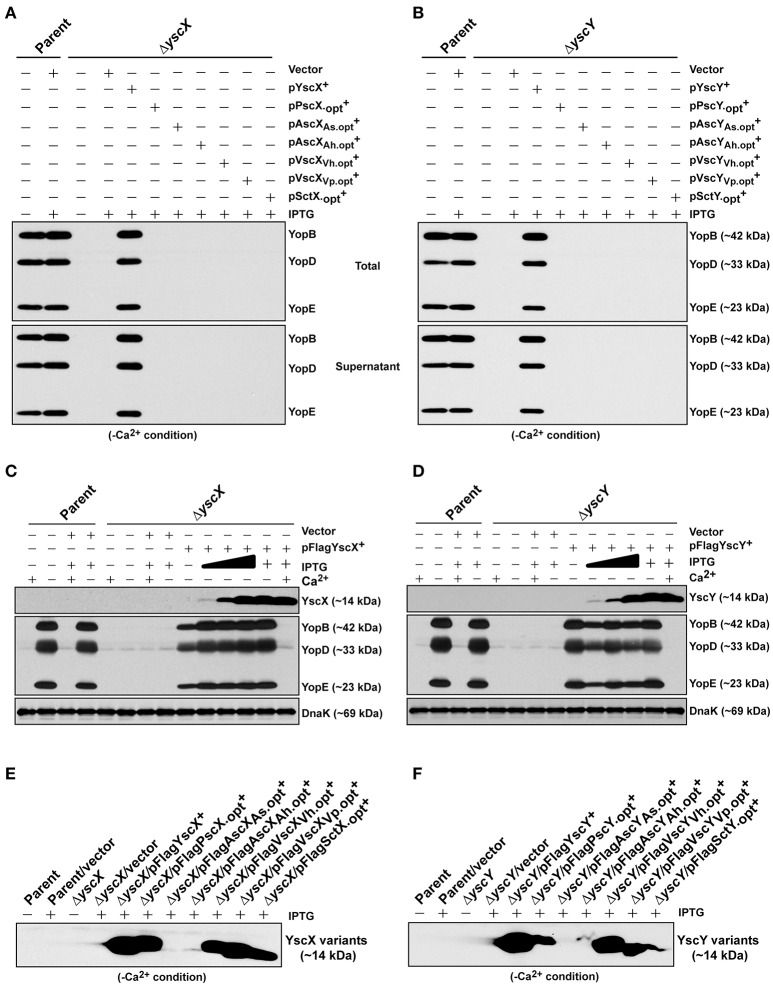
Assessing functional complementation by codon-optimized *yscX*- and *yscY*-like alleles. Synthesized Yop's associated with total fractions (proteins contained within intact bacteria and secreted to the culture medium) or with supernatant (secreted free to the extracellular medium) were analyzed in Δ*yscX* null-mutant complemented with YscX and codon-optimized YscX-like protein family members **(A)** or in Δ*yscY* null-mutant complemented with YscY and codon-optimized YscY-like protein family **(B)**. Polyclonal anti-YopB, anti-YopD and anti-YopE were used for detection of Yops. The “+” symbol indicates the addition of IPTG to a final concentration of 0.4 mM. Proteins associated with bacterial pellet from *Y. pseudotuberculosis* cultures harboring either pFLAG-YscX **(C)** or pFLAG-YscY **(D)** grown in secretion-permissive condition (absence of Ca^2+)^ and supplemented with increasing concentration of IPTG were analyzed by Western immunoblot using anti-FLAG™, anti-YopB, anti-YopD, anti-YopE, and anti-DnaK. The “−” symbol indicates absence of a particular component, while the “+” symbol indicates the presence of a particular component. In the case of IPTG, the “+” symbol indicates a final concentration of 0.4 mM IPTG was added, while the filled in graduation symbol reflects an incremental increase of IPTG according to the following final concentrations of 0.01, 0.02, and 0.1 mM. Steady state accumulation in the bacterial pellet of YscX protein family **(E)** and YscY protein family **(F)** produced from codon-optimized alleles following growth of *Y. pseudotuberculosis* in secretion-permissive conditions (absence of Ca^2+^). Protein was fractionated by SDS-PAGE and detected by immunoblot using monoclonal anti-FLAG™ antiserum. The “−” symbol indicates bacterial growth in the absence of IPTG, while “+” symbol indicates a final concentration of 0.4 mM was added. Strains: Parent (YPIII/pIB102); Complemented YPIII/pIB102, pMMB208 (Vector);Δ*yscX* (YPIII/pIB880); complemented YPIII/pIB880, pMMB208 (Vector); complemented YPIII/pIB880, pJMG242 (FLAG™-YscX^+^); complemented YPIII/pIB880, pJMG261(FLAG™-${\text{PscX.}}_{\text{opt}}^{+}$); complemented YPIII/pIB880, pJMG262 (FLAG™-${\text{AscX}}_{\text{As}.\text{opt}}^{+}$); complemented YPIII/pIB880, pJMG263 (FLAG™-${\text{AscX}}_{\text{Ah}.\text{opt}}^{+}$); complemented YPIII/pIB880, pJMG264 (FLAG™-${\text{VscX}}_{\text{Vh}.\text{opt}}^{+}$); complemented YPIII/pIB880, pJMG265 (FLAG™-${\text{VscX}}_{\text{Vp}.\text{opt}}^{+}$); complemented YPIII/pIB880, pMF266 (FLAG™-${\text{SctX.}}_{\text{opt}}^{+}$); Δ*yscY* (YPIII/pIB890); complemented YPIII/pIB890, pMMB208 (Vector); complemented YPIII/pIB890, pMF800 (FLAG™-YscY^+^); complemented YPIII/pIB890, pJMG267 (FLAG™-PscY.opt^+^); complemented YPIII/pIB890, pJMG268 (FLAG™-${\text{AscY}}_{\text{As}.\text{opt}}^{+}$); complemented YPIII/pIB890, pJMG269 (FLAG™-${\text{AscY}}_{\text{Ah}.\text{opt}}^{+}$); complemented YPIII/pIB890, pJMG270 (FLAG™-${\text{VscY}}_{\text{Vh}.\text{opt}}^{+}$); complemented YPIII/pIB890, pJMG271 (FLAG™-${\text{VscY}}_{\text{Vp}.\text{opt}}^{+}$); complemented YPIII/pIB890, pJMG272 (FLAG™-${\text{SctY}}_{.\text{opt}}^{+}$); complemented YPIII/pIB880, pJEB291 (YscX^+^); complemented YPIII/pIB880, pJMG293 (${\text{PscX.}}_{\text{opt}}^{+}$); complemented YPIII/pIB880, pJMG294 (${\text{AscX}}_{\text{As}.\text{opt}}^{+}$); complemented YPIII/pIB880, pJMG295 (${\text{AscX}}_{\text{Ah}.\text{opt}}^{+}$); complemented YPIII/pIB880, pJMG296 (${\text{VscX}}_{\text{Vh}.\text{opt}}^{+}$); complemented YPIII/pIB880, pJMG297 (${\text{VscX}}_{\text{Vp}.\text{opt}}^{+}$); complemented YPIII/pIB880, pJMG298 (${\text{SctX}}_{.\text{opt}}^{+}$); YPIII/pIB890, pJEB292 (YscY^+^); YPIII/pIB890, pJMG286 (${\text{PscY.}}_{\text{opt}}^{+}$); YPIII/pIB890, pJMG287 (AscY_As_$._{\text{opt}}^{+}$); YPIII/pIB890, pJMG288 (AscY_Ah_$._{\text{opt}}^{+}$); YPIII/pIB890, pJMG289 (VscY_Vh_${\text{.}}_{\text{opt}}^{+}$); YPIII/pIB890, pJMG290 (VscY_Vp_$._{\text{opt}}^{+}$); YPIII/pIB890, pJMG291 (${\text{SctY.}}_{\text{opt}}^{+}$). Molecular mass values shown in parentheses were deduced from primary amino acid sequences.

Interestingly, an analysis of codon usage scores in the *yscX* and *yscY*-like gene families based on the codon adaption index (CAI) revealed the presence of codon usage biases of different genes (Figure S4). Hence, we synthesized commercially a series of synthetic alleles that were optimized for production in *Y. pseudotuberculosis* by actively considering (i) codon usage bias, (ii) GC content, and (iii) mRNA secondary structure. In all cases the decoded amino acid sequence remained identical to that encoded by the native gene sequences. In the optimized synthetic genes, the number of unfavorable codons was reduced by upgrading the CAI value to ~0.9 and by adjusting the GC content more closely resembling that of *Yersinia* (Figures S4, S5). Predicted mRNA secondary structure was not affected by optimization (data not shown). To assess protein production, we cloned both native and synthetically optimized gene sequences as N-terminal FLAG™-tagged variants into pMMB208 vector under the control of an IPTG-inducible promoter. Following introduction into *Y. pseudotuberculosis* and subsequent growth in BHI minus Ca^2+^, bacterial pellets standardized against bacterial cell number were examined for accumulation of FLAG™-tagged protein. Except for the SctX and VscY_Vh_ variants, codon optimization led to improved production of all the YscX-like and YscY-like variants with accumulated levels ranging between 1.3- and 7.0-fold more abundant then their native (non-optimized) counterparts (Figure S6). Interestingly, despite the successful efforts to codon optimize, accumulated levels of some recombinant proteins such as those originally derived from *Aeromonas* sp. still failed to reach a level close to that achieved for the native *Yersinia* proteins (Figure 6).

Significantly, this low level protein production was not due to an inferior transcriptional output because accumulated mRNA levels transcribed from all *yscX*-like (Figure S7A) and *yscY*-like (Figure S7B) genes was similar, which is consistent with transcription being driven by an identical promoter and transcriptional start site architecture in every case. Furthermore, the stability of accumulated *ascX* mRNA transcripts (Figure S7C) and *ascY* mRNA transcripts (Figure S7D) was equivalent to *yscX* and *yscY* mRNA transcripts. Thus, additional hitherto unknown regulatory elements must be limiting AscX and AscY production in *Y. pseudotuberculosis*.

Nevertheless, given that in most cases elevated production arose from codon optimization, we assessed if these codon optimized YscX-like and YscY-like protein variants could restore T3S to the Δ*yscX* null mutant and Δ*yscY* null-mutant, respectively. The optimized *yscX*-like and *yscY*-like genes were cloned individually into pMMB67EHgm and Yops production associated with total bacterial culture and Yops secretion associated with bacterial supernatants were monitored in secretion permissive growth conditions. Once again however, Yops production and secretion could be restored only when the Δ*yscX* null mutant was complemented with native YscX protein (Figure 6A) and Δ*yscY* null mutant with native YscY protein (Figure 6B).

To be sure that all other codon-optimized homologs were sufficiently produced to permit complementation, we compared these levels to the minimal amount of YscX and YscY sufficient to restore T3S to the Δ*yscX* and Δ*yscY* null mutants respectively. Critically, an undetectable amount of native YscX and YscY ectopically produced during growth in the presence of 0.02 mM of IPTG was already sufficient to restore high capacity T3S to the Δ*yscX* null mutant (Figure 6C) and Δ*yscY* null mutant (Figure 6D), respectively. These levels were quite noticeably much less than accumulated amounts detected for all of the non-complementing YscX-like (Figure 6E) and YscY-like (Figure 6F) variants when bacteria were grown in the presence of 0.4 mM IPTG. From this data we infer that insufficient expression of non-native YscX- and YscY-like family members cannot explain their inability to function in *Y. pseudotuberculosis*.

### Codon optimized alleles do not suppress a functional *Yersinia* T3SS

**Figure 7:**
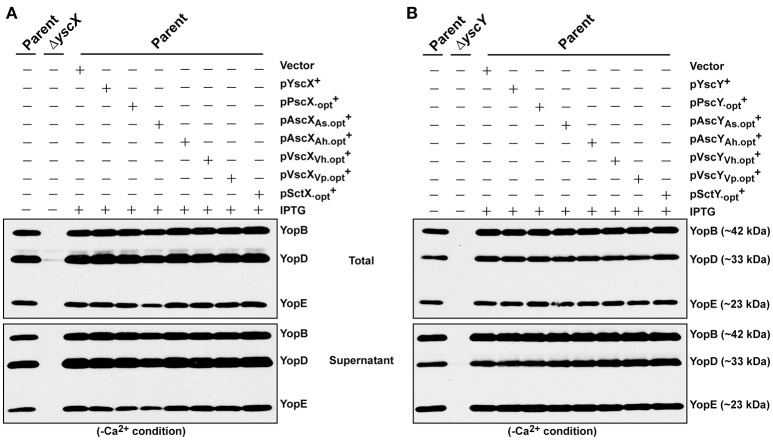
Assessing YscX-like and YscY-like homolog function in the presence of a fully intact type III secretion system. Bacteria were grown in BHI medium under secretion-permissive condition (absence of Ca^2+^). Proteins contained within intact bacteria and secreted to the culture medium (Total) or secreted free into the extracellular medium (Supernatant) were fractionated on a 12% SDS-PAGE and analyzed by immunoblotting using polyclonal rabbit anti-YopB, anti-YopD, and anti-YopE antiserum. IPTG was added to a final concentration of 0.4 mM where indicated. Strains: Parent (YPIII/pIB102); Δ*yscX* (YPIII/pIB880); Complemented YPIII/pIB102, pMMB67EHgm (Vector); Complemented YPIII/pIB102, pJEB291 (YscX^+^); complemented YPIII/pIB102, pJMG293 (${\text{PscX.}}_{\text{opt}}^{+}$); complemented YPIII/pIB102, pJMG294 (${\text{AscX}}_{\text{As}.\text{opt}}^{+}$); complemented YPIII/pIB102, pJMG295 (${\text{AscX}}_{\text{Ah}.\text{opt}}^{+}$); complemented YPIII/pIB102, pJMG296 (${\text{VscX}}_{\text{Vh}.\text{opt}}^{+}$); complemented YPIII/pIB102, pJMG297 (${\text{VscX}}_{\text{Vp}.\text{opt}}^{+}$); complemented YPIII/pIB102, pJMG298 (${\text{SctX}}_{.\text{opt}}^{+}$); Δ*yscY* (YPIII/pIB890); YPIII/pIB102, pJEB292 (YscY^+^); YPIII/pIB102, pJMG286 (${\text{PscY.}}_{\text{opt}}^{+}$); YPIII/pIB102, pJMG287 (AscY_As_$._{\text{opt}}^{+}$); YPIII/pIB102, pJMG288 (AscY_Ah_$._{\text{opt}}^{+}$); YPIII/pIB102, pJMG289 (VscY_Vh_$._{\text{opt}}^{+}$); YPIII/pIB102, pJMG290 (VscY_Vp_$._{\text{opt}}^{+}$); YPIII/pIB102, pJMG291 (${\text{SctY.}}_{\text{opt}}^{+}$). Molecular mass values shown in parentheses were deduced from primary amino acid sequences.

Despite the YscX- and YscY-like homologs being unable to substitute for the respective loss of *yscX* or *yscY* in *Y. pseudotuberculosis*, we still wondered if they possess any activity at all when expressed in *Y. pseudotuberculosis*. To assess this, we examined for the ability of produced homologs to exert a dominant negative effect on *Yersinia* T3SS. All of the codon-optimized homologs of YscX and YscY under control of the IPTG inducible promoter of pMMB67EHgm were introduced in parental *Y. pseudotuberculosis* harboring an intact and fully functional T3SS. Firstly, we examined the protein expression profile of YscX and YscY family members in parental *Yersinia* background and found them to be comparable to what we had previously observed in the knockout strain backgrounds (data not shown). We then assessed the impact of this production on T3SS function as measured by Yops production and secretion. Significantly, neither the production of codon-optimized YscX-like (Figure 7A) nor YscY-like (Figure 7B) homologs interfered with T3SS activity by parental bacteria. The complete absence of any dominant negative effect implies that none of the YscX-like or YscY-like proteins are recognized by the *Yersinia* T3SS, which underscores that these proteins are truly non-functional in *Yersinia* bacteria. Taken altogether, our data indicates that an undisclosed feature(s) inherent in the native YscX and YscY products is necessary for proper T3S function in *Yersinia*, and one or more of these features cannot be substituted for by a genetically related gene from another bacterial source.

## Discussion

On the basis that a number of core T3SS basal body components associated with bacterial inner and outer membranes are highly conserved, these have been the focus of studies to establish at least eight phylogenetically distinct T3SS sub-families among sequenced bacteria (Troisfontaines and Cornelis, 2005; Abby and Rocha, 2012). In addition, there exist critical components that are present only in a restricted T3SS sub-family. For example, the poorly studied proteins YscX and YscY exist only in the Ysc T3SS sub-family, and the prototype of this family is the plasmid encoded Ysc-Yop system of human pathogenic species of *Yersinia*. In this study, we have reported on the evolutionary conservation among the YscX and YscY protein families. We also explored their function in two ways. The first approach examined the binary interactions between all YscX-like and YscY-like family members, and the ability of this YscX-YscY complex to interact with the core basal body component YscV. The second approach examined the ability of all YscX-like and YscY-like family members to complement a type III secretion defect in *Y. pseudotuberculosis* brought about by a deletion of *yscX* and/or *yscY*. Our focus was on six different homologs sourced from *P. aeruginosa, A. hydrophila, A. salmonicida, V. harveyi, V. parahaemolyticus* and *P. luminescens*. Despite showing robust and interchangeable binary YscX-YscY interactions and ternary YscX-YscY-YscV interactions, none of the homologs could restore T3S to *Y. pseudotuberculosis* lacking functional YscX and/or YscY. Thus, it seems that this YscX-YscY-YscV system is functionally conserved in Ysc-like T3SSs, but that each set exhibits functional specificity to assemble their own apparatus, and this could include recognition of their own set of substrates.

Events allowing YscX to form stable complexes with the known cognate chaperone YscY, and for the YscX-YscY complex to interact with the core T3SS structural component YscV, are considered to constitute integral elements of T3SS assembly and function (Day and Plano, 2000; Diepold et al., 2012). Our n-hybrid analyses suggests that these interactions are a key attribute of all YscX-like, YscY-like and YscV-like homologs, serving an important T3SS assembly function that is evolutionarily conserved. In fact, the need to form a bipartite and a tripartite interaction is most likely an ancient role of these protein families, and maybe the reason why YscX and YscY could functionally substitute for the absence of PscX and PscY in mutants of *P. aeruginosa* (Bröms et al., 2005). However, merely having the ability to establish these known reciprocal interactions was clearly not enough to permit any of the homologs to mediate functional exchange in *Y. pseudotuberculosis*. Indeed this suggests that both YscX and YscY possess other molecular targets that are arguably unique to Ysc-Yop T3SS function. An approach to identify these putative targets within soluble bacterial lysate or solubilized bacterial membrane fractions would be to combine pull down technology using the Flag-YscX or Flag-YscY as a bait with mass spectroscopy detection. We can only assume that this need for additional molecular interactions arose as a consequence of *Yersinia* evolving the ability to thrive in a particular ecological niche. This notion is consistent with previous findings that correlate a diversification of bacterial T3SSs with known bacterial-host interactions (Abby and Rocha, 2012).

Sequence alignments revealed several obvious regions of uniqueness within the YscX and YscY amino acid sequence, respectively. At first glance, these are too numerous to perform targeted mutagenesis to screen for loss-of-function in the *Yersinia* proteins or gain-of-function in the heterologous proteins. Hence, we are now attempting to first map potentially critical functional domains unique to YscX and YscY, prior to embarking on a site-directed mutagenesis strategy. A series of chimeric alleles between *yscX* and *yscX*-like homologs as well as *yscY* and *yscY*-like homologs are being created. We proposed that by generating large N-terminal and C-terminal chimeric sets that contain progressively more native YscX sequence and native YscY sequence presents an alternative tool to define a minimal genetic content that confers to the chimera a functional competence for *Y. pseudotuberculosis* Ysc-Yop T3SS activity, which can then be subsequently confirmed using site-directed mutagenesis.

It is likely that such sequence would serve as an interaction interface for engaging with one or more unknown targets, making it useful as a bait to identify this unique contributor to T3SS activity in *Y. pseudotuberculosis*. Some unique non-reciprocal interactions are already known—YscY interacts with the translocator class chaperone LcrH/SycD in *Y. pseudotuberculosis* (Francis et al., 2001; Bröms et al., 2005), while PscY (termed Pcr4) interacts with Pcr1 (the equivalent of TyeA in *Yersinia*) in *P. aeruginosa* (Yang et al., 2007). However, neither of these two interactions are prerequisites for the physical assembly of their respective T3SSs, but rather function primarily in the fine-tuning of substrate secretion control. In particular, LcrH/SycD influences the ability of YopD to bind both *yop* mRNA and the 30S ribosomal subunit to modulate translation initiation of *yop* mRNA and accelerate its turnover (Anderson et al., 2002; Chen and Anderson, 2011; Kopaskie et al., 2013). The consequence of this LcrH-dependent YopD RNA binding activity is to post-transcriptionally regulate T3SS genes to impart a level of hierarchal secretion control. Significantly, a strain defective in LcrH does not loose capacity to secrete substrates (Wattiau et al., 1994; Francis et al., 2001; Edqvist et al., 2006), and neither do defined mutants in LcrH that are unable to interact with YscY (Francis et al., 2001; Bröms et al., 2005). Thus, LcrH-YscY complex formation is likely to feed into the post-transcriptionally active complex of YopD, LcrH, and *yop* mRNA with the sole purpose to further fine-tune temporal and spatial control of substrate secretion. It is not anticipated to assist in assembly of a secretion-competent apparatus *per se*. On this basis, we do not believe that the specificity of LcrH-YscY interaction in *Yersinia* is the reason why heterologous protein expression cannot support T3S. Hence, we will continue to pursue evidence for a unique non-reciprocal interaction involving YscX and YscY that is essential for the physical assembly of the Ysc-Yop T3SS, rather than one involved in substrate secretion control.

Through sequence alignments we did observe that only native YscX contained two cysteine residues in codon positions that were absolutely conserved. At this stage we do not know if these cysteine residues are reduced or participate in inter- or intra-disulfide bond formation. If they do participate in disulfide bond formation, this would be a unique feature of native YscX, and gives a reason as to why heterologous YscX-like proteins that lack these two conserved cysteines are not functional in *Yersinia*. It is well-established that disulfide bonds are an important structural element of the secretome of bacterial pathogens (De Geyter et al., 2016; Bocian-Ostrzycka et al., 2017). This is true also for human pathogenic *Yersinia* sp., where a role for thiol bonding is reported for the Ysc-Yop T3SS, the Caf1 chaperone-usher system, and the invasin autotransporter (Leong et al., 1993; Zav'yalov et al., 1997; Jackson and Plano, 1999; Mitchell et al., 2017). Thus, a logical next step is to use site-directed mutagenesis to investigate possible inter- or intra-disulfide bond formation in YscX.

Following completion of the needle assembly in *Y. enterocolitica*, YscX secretion into the extracellular milieu occurs (Diepold et al., 2012). However, the purpose of YscX secretion is enigmatic. Our ongoing studies have so far revealed that secretion of YscX is absolutely required for a functional T3SS in *Y. pseudotuberculosis* (unpublished data). One reason for YscX secretion might be to free up a complex of YscY-YscV to recognize and secrete other later subsets of T3S substrates, namely the translocators that are necessary to trigger the next stage of injectisome assembly and the host-modulating effectors that are injected into eukaryotic cells (Dewoody et al., 2013). In parallel, secreted YscX might comprise a minor component of the external needle, although experimental evidence to support this is completely lacking. Alternatively, secreted YscX may comprise a structural component within the needle assembly platform, such as via a cooperation with the YscI inner rod. Hence, to understand the role of YscX secretion in T3SS function is important. This pursuit is made all the more intriguing on the basis that PscY is the secreted component of the T3SS of *P. aeruginosa*, not PscX as one may have expected (Yang et al., 2007).

Given that all indicators point to the fact that YscX secretion is essential for T3SS function in *Yersinia*, it follows that a functional requirement for complementation of the Δ*yscX* deletion mutant would likely be secretion of the corresponding YscX-like homologs. Taking account of the already stated PscY secretion exception (Yang et al., 2007), we are aware of only one other YscX-like homolog that has been proven experimentally to be a T3SS substrate—the AscX substrate of *A. salmonicida* (Vanden Bergh et al., 2013). We attempted to examine secretion of all YscX-like proteins by parental *Y. pseudotuberculosis* containing a fully functional Ysc-Yop T3SS. However, this was complicated by the need to produce all YscX-like variants as a FLAG™ fusion to facilitate their immune-detection, for this tag may impact on fusion protein secretion efficiency. Indeed, the amount of FLAG™-YscX secreted was only a fraction of the amount that was produced (data not shown). Furthermore, we could not corroborate the findings of Vanden Bergh et al. (2013), for the yields of AscX were routinely too low to assess its secretion. Consistent with this, most other FLAG™-tagged variants were not even detected in the secretion fraction, including PscX that is not a T3S substrate (Yang et al., 2007) (data not shown). The exception to this was the observation that FLAG™-VscX_Vp_ and FLAG™-SctX were active substrates of a fully intact Ysc-T3SS of *Y. pseudotuberculosis* (data not shown). Hence, at present we cannot establish a clear correlation between lack of secretion and lack of functional complementation.

One final observation of interest stemming from this study concerned the poor production of the two AscX proteins and the two AscY proteins in the *Y. pseudotuberculosis* background. This occurred despite (1) YscX and AscX as well as YscY and AscY sharing a sequence identity above the 50 percentile, (2) an abundance of comparatively stable mRNA being initiated from an identical promoter used to support the expression of all other genes studied in this report, and (3) the use of synthetic genes that had been codon optimized to maximize efficient translation in *Y. pseudotuberculosis*. Hence, some hitherto unknown phenomenon is responsible for the lack of heterologous production of these proteins in *Yersinia*. A straightforward reason for this is that they are normally stabilized by species-specific protein–protein interactions. The YscY chaperone is thought to function as an YscX stabilizer (Iriarte and Cornelis, 1999; Day and Plano, 2000). Indeed, this motivated the dual expression complementation experiment performed in this study. However, co-expression of AscX with cognate AscY did not increase the production of either protein. A conclusion from this work is that AscY alone is probably insufficient for AscX stabilization, meaning that the complex of AscX-AscY requires stabilizing influence from additional protein–protein interactions not supported by expression in a heterologous host like *Yersinia*. Another reason for poor AscX and AscY yield could also be that some aspect of mRNA sequence and/or structure defined by the *ascX* and *ascY* open reading frames influences that rate of their translation in *Yersinia*. As a means to pin-point the reason for low yield, we are currently analyzing allelic chimeras between *ascX* and *yscX* as well as *ascY* and *yscY*. This approach has the possibility to locate the genetic regions in AscX and AscY that are either (1) the interface for species-specific protein–protein interactions that are necessary for protein stability, or (2) the site of novel posttranscriptional control mechanisms that have potential to influence the spatial and temporal control of T3SS.

In summary, on the basis of complementation assays measuring the capacity for *in vitro* synthesis and secretion of Yops, it appears that the function of native YscX and YscY is evolutionary optimized to contribute unique and essential information for the physical assembly of the Ysc-Yop T3SS by pathogenic *Yersinia* sp. Thus, the challenge that lies ahead is to define the function of both YscX and YscY, and to decipher whether this function requires the recruitment of additional molecular components. In contrast, from this work we now know that the established bipartite YscX-YscY interaction and the tripartite YscX-YscY-YscV interaction is not a unique feature restricted only to native components of *Yersinia*. Rather, these interactions are preserved among all heterologous YscX-like and YscY-like family members, and this probably reflects an ancient and evolutionarily conserved universal function of these proteins.

## Author contributions

JG, AA, MKF, TC, and MSF conceived and planned the experiments. SC and MSF contributed essential materials. JG, AA, MKF, and TC carried out the experiments and contributed to sample preparation. JG, AA, MKF, TC, SC, AZ, and MSF contributed to the interpretation of the results. JG and MSF took the lead in writing the manuscript. All authors provided critical feedback and helped shape the research, analysis, and manuscript.

### Conflict of interest statement

The authors declare that the research was conducted in the absence of any commercial or financial relationships that could be construed as a potential conflict of interest.
