# Supplementary material for: Heterologous Complementation Studies With the YscX and YscY Protein Families Reveals a Specificity for Yersinia pseudotuberculosis Type III Secretion
Source: Front Cell Infect Microbiol. 2018 Mar 16;8:80. doi: 10.3389/fcimb.2018.00080 (PMC5864894; doi:10.3389/fcimb.2018.00080)
Supplement: Supplementary file 1 [file DataSheet1.PDF]

Electronic supplementary material

Heterologous complementation studies with the YscX and YscY protein families reveals a specificity for *Yersinia pseudotuberculosis* type III secretion

Jyoti M. Gurung[1,2], Ayad A. A. Amer[1,2], Monika K. Francis[1,2], Tiago R. D. Costa[1,2,5], Shiyun Chen[3], Anton V. Zavialov[4], and Matthew S. Francis[1,2]

[1]Department of Molecular Biology, Umeå University, Umeå, Sweden
[2]Umeå Centre for Microbial Research, Umeå University, Umeå, Sweden
[4]Key Laboratory of Special Pathogens and Biosafety, Wuhan Institute of Virology, Chinese Academy of Sciences Wuhan, China
[4]Department of Chemistry, University of Turku, Turku, Finland
[5]MRC Centre for Molecular Bacteriology and Infection, Department of Life Sciences, Imperial College London, London SW7 2AZ, United Kingdom

TABLES:

Table S1| Strains and plasmids used in this study
Table S2| Oligonucleotides used in this study

FIGURES:

Figure S1| Amino acid sequence alignment among YscX and YscY protein families
Figure S2| Reciprocal interactions between YscX family members and YscY family members generated in the yeast two-hybrid assay
Figure S3| Protein expression profiles of native YscX and YscY and their homologues in *Y. pseudotuberculosis*
Figure S4| Codon-usage bias adjustment of *yscX*-like alleles and *yscY*-like alleles
Figure S5| Comparison of the GC content between native and codon-optimized *yscX*-like alleles and *yscY*-like alleles
Figure S6| Relative protein levels of accumulated native and optimized YscX and YscY homologues in *Y. pseudotuberculosis*
Figure S7| Analysis of gene transcription by qualitative RT-PCR

Table S1| Strains and plasmids used in this study

Strain or plasmid	Relevant genotype or phenotype	Source or reference
**STRAIN**		
E. coli		
TOP10	F^{-}*mcrA*, Δ(*mrr-hsdRMS-mcrBC*), Φ80*lacZ*ΔM15, Δ*lacX*74, *recA1*, *deoR*, *araD*139, Δ(*ara-leu*)7679, *galU, galK, rpsL* (Str^{R}), *endA1*, *nupG*	Invitrogen
DH5	F^{-}, *recA*1, *endA*1, *hsdR*17, *supE*44, *thi*-1, *gyrA*96, *relA*1	(Hanahan, 1985)
S17-1λ*pir*	*recA*, *thi*, *pro*, *hsdR*$^{-}$*M*$^{+}$, Sm^{R}, <RP4:2-Tc:Mu:Ku:Tn7>Tp^{R}	(Simon et al., 1983)
Y. pseudotuberculosis		
YPIII/pIB102	*yadA*::Tn5, Km^{R} (wild type)	(Bölin and Wolf-Watz, 1984)
YPIII/pIB880	pIB102, *yscX* in frame deletion of codons 24-106	(Bröms et al., 2005)
YPIII/pIB890	pIB102, *yscY* in frame deletion of codons 14-90	(Bröms et al., 2005)
YPIII/pIB881	pIB102, *yscX, yscY* in frame deletion spanning from codons 24 of *yscX* to 90 of *yscY*	(Bröms et al., 2005)
Y. enterocolitica		
W22703/pYVe227	Spontaneous Nal^{R} isolate of W227, serotype 0:9	Guy Cornelis
A. hydrophilia		
AH3	Clinical isolate, serotype O34 (wild type)	Juan Tomás
A. salmonicida		
JF2267	Pathogenic isolate from artic char (wild type)	Joachim Frey
P. aeruginosa		
PAK	wild type clinical isolate	(Bradley, 1974)
PAK*pcr3*	PAK, *pcr3* in frame deletion of codons 7-116	(Bröms et al., 2005)
PAK*pcr4*	PAK, *pcr4* in frame deletion of codons 7-101	(Bröms et al., 2005)
P. luminescens		
TT01 (Rif)	Spontaneous Rif^{R} isolate of wild type TT01	David Clarke
V. harveyi		
BB120	Also known as ATCC BAA-1116 (wild type)	Debra Milton
V. parahaemolyticus		
RIMD2210633	Clinical isolate, serotype O3:K6 (wild type)	Tetsuya Iida
S. cerevisiae		
AH109	*MATa, trp1-901, leu2-3, 112, ura3-52, his3-200, gal4Δ, gal80Δ, LYS2 : : GAL1*$_{UAS}$*-GAL1*$_{TATA}$*-HIS3, GAL2*$_{UAS}$*-GAL2*$_{TATA}$*-ADE2, URA3 : : MEL1*$_{UAS}$*-MEL1* $_{TATA}$*-lacZ*	Clontech Laboratories
Y190	*MATa, ura3-52, his3-200, ade2-101, lys2-801, trp1-901, leu2-3, 112, gal4Δ, gal80Δ, cyh*^{r}*2, LYS2 : : GAL1*$_{UAS}$*-HIS3*$_{TATA}$*-HIS3, MEL1 URA3 : : GAL1*$_{UAS}$*-GAL1*$_{TATA}$*-lacZ*	Clontech Laboratories
**PLASMID**		
pMMB67EHgm	p*tac* expression vector, Gm^{R}	(Fürste et al., 1986)
pJEB291	*Eco*RI/*Hind*III PCR fragment of *yscX* in pMMB67EHgm, Gm^{R}	(Bröms et al., 2005)

pJEB292	*Eco*RI/*Pst*I PCR fragment of *yscY* in pMMB67EHgm, Gm^R	(Bröms et al., 2005)
pJEB295	*Eco*RI/*Hind*III PCR fragment of *pscX* (*pcr3*) in pMMB67EHgm, Gm^R	(Bröms et al., 2005)
pJEB296	*Eco*RI/*Hind*III fragment of *pscY* (*pcr4*) in pMMB67EHgm, Gm^R	(Bröms et al., 2005)
pMF720	*Eco*RI/*Bam*HI fragment of *ascX* (*A. salmonicida*) in pMMB67EHgm, Gm^R	This study
pMF721	*Eco*RI/*Bam*HI fragment of *ascY* (*A. salmonicida*) in pMMB67EHgm, Gm^R	This study
pMF722	*Eco*RI/*Bam*HI fragment of *ascX* (*A. hydrophilia*) in pMMB67EHgm, Gm^R	This study
pMF723	*Eco*RI/*Bam*HI fragment of *ascY* (*A. hydrophilia*) in pMMB67EHgm, Gm^R	This study
pMF724	*Eco*RI/*Bam*HI fragment of *vscX* (*V. harveyi*) in pMMB67EHgm, Gm^R	This study
pMF796	*Bam*HI/*Pst*I fragment of *vscY* (*V. harveyi*) in pMMB67EHgm, Gm^R	This study
pMF725	*Eco*RI/*Bam*HI fragment of *vscX* (*V. parahaemolyticus*) in pMMB67EHgm, Gm^R	This study
pMF726	*Eco*RI/*Bam*HI fragment of *vscY* (*V. parahaemolyticus*) in pMMB67EHgm, Gm^R	This study
pMF727	*Eco*RI/*Bam*HI fragment of *sctX* in pMMB67EHgm, Gm^R	This study
pMF728	*Eco*RI/*Bam*HI fragment of *sctY* in pMMB67EHgm, Gm^R	This study
pMF847	*Eco*RI/*Bam*HI PCR fragment of *yscX* (*Y. enterocolitica*) in pMMB67EHgm, Gm^R	This study
pJEB335	*Eco*RI/*Hind*III PCR fragment of *pscX* and *pscY* (*pcr3* and *pcr4*) in pMMB67EHgm, Gm^R	(Bröms et al., 2005)
pJEB340	*Eco*RI/*Pst*I PCR fragment of *yscX* and *yscY* in pMMB67EHgm, Gm^R	(Bröms et al., 2005)
pMF733	*Eco*RI/*Bam*HI PCR fragment of *ascX* and *ascY* (*A. salmonicida*) in pMMB67EHgm, Gm^R	This study
pMF734	*Eco*RI/*Bam*HI PCR fragment of *ascX* and *ascY* (*A. hydrophilia*) in pMMB67EHgm, Gm^R	This study
pMF735	*Eco*RI/*Bam*HI PCR fragment of *vscX* and *vscY* (*V. parahaemolyticus*) in pMMB67EHgm, Gm^R	This study
pMF736	*Eco*RI/*Bam*HI PCR fragment of *sctX* and *sctY* in pMMB67EHgm, Gm^R	This study
pMF797	*Bam*HI/*Pst*I PCR fragment of *vscX* and *vscY* (*V. harveyi*) in pMMB67EHgm, Gm^R	This study
pJMG293	*Eco*RI/*Bam*HI PCR fragment of codon-optimized *pscX* (*P. aeruginosa*) in pMMB67EHgm, Gm^R	This study
pJMG286	*Eco*RI/*Bam*HI PCR fragment of codon-optimized *pscY* (*P. aeruginosa*) in pMMB67EHgm, Gm^R	This study
pJMG294	*Eco*RI/*Bam*HI PCR fragment of codon-optimized *ascX* (*A. salmonicida*) in pMMB67EHgm, Gm^R	This study
pJMG287	*Eco*RI/*Bam*HI PCR fragment of codon-optimized *ascY* (*A. salmonicida*) in pMMB67EHgm, Gm^R	This study
pJMG295	*Eco*RI/*Bam*HI PCR fragment of codon-optimized *ascX* (*A. hydrophila*) in pMMB67EHgm, Gm^R	This study
pJMG288	*Eco*RI/*Bam*HI PCR fragment of codon-optimized *ascY* (*A. hydrophila*) in pMMB67EHgm, Gm^R	This study
pJMG296	*Eco*RI/*Bam*HI PCR fragment of codon-optimized *vscX* (*V. harveyi*) in pMMB67EHgm, Gm^R	This study
pJMG289	*Bam*HI/*Pst*I PCR fragment of codon-optimized *vscY* (*V. harveyi*) in pMMB67EHgm, Gm^R	This study

pJMG297	*Eco*RI/*Bam*HI PCR fragment of codon-optimized *vscX* (*V. parahaemolyticus*) in pMMB67EHgm, Gm^R	This study
pJMG290	*Eco*RI/*Bam*HI PCR fragment of codon-optimized *vscY* (*V. parahaemolyticus*) in pMMB67EHgm, Gm^R	This study
pJMG298	*Eco*RI/*Bam*HI PCR fragment of codon-optimized *sctX* (*P. luminescens)* in pMMB67EHgm, Gm^R	This study
pJMG291	*Eco*RI/*Bam*HI PCR fragment of codon-optimized *sctY* (*P. luminescens)* in pMMB67EHgm, Gm^R	This study
pMMB208	p*tac* expression vector, Cm^R	(Morales et al., 1991)
pJMG242	*Bam*HI/*Eco*RI PCR fragment of 5-prime FLAG™-*yscX* in pMMB208, Cm^R	This study
pJMG243	*Bam*HI/*Eco*RI PCR fragment of 5-prime FLAG™-*pscX* (*P. aeruginosa*)in pMMB208, Cm^R	This study
pJMG244	*Bam*HI/*Eco*RI PCR fragment of 5-prime FLAG™-*ascX* (*A. salmonicida*) in pMMB208, Cm^R	This study
pJMG245	*Bam*HI/*Eco*RI PCR fragment of 5-prime FLAG™-*ascX* (*A. hydrophila*)in pMMB208, Cm^R	This study
pJMG246	*Bam*HI/*Eco*RI PCR fragment of 5-prime FLAG™-*vscX* (*V. harveyi*) in pMMB208, Cm^R	This study
pJMG247	*Bam*HI/*Eco*RI PCR fragment of 5-prime FLAG™-*vscX* (*V. parahaemolyticus*) in pMMB208, Cm^R	This study
pJMG248	*Bam*HI/*Eco*RI PCR fragment of 5-prime FLAG™-*sctX* (*P. luminescens*) in pMMB208, Cm^R	This study
pJMG261	*Bam*HI/*Eco*RI PCR fragment of codon-optimized 5-prime FLAG™-*pscX* (*P. aeruginosa*) in pMMB208, Cm^R	This study
pJMG262	*Bam*HI/*Eco*RI PCR fragment of codon-optimized 5-prime FLAG™-*ascX (A. salmonicida)* in pMMB208, Cm^R	This study
pJMG263	*Bam*HI/*Eco*RI PCR fragment of codon-optimized 5-prime FLAG™-*ascX (A. hydrophila)* in pMMB208, Cm^R	This study
pJMG264	*Bam*HI/*Eco*RI PCR fragment of codon-optimized 5-prime FLAG™-*vscX (V. harveyi)* in pMMB208, Cm^R	This study
pJMG265	*Bam*HI/*Eco*RI PCR fragment of codon-optimized 5-prime FLAG™-*vscX (V. parahaemolyticus)* in pMMB208, Cm^R	This study
pJMG266	*Bam*HI/*Eco*RI PCR fragment of codon-optimized 5-prime FLAG™-*sctX (P. luminescens)* in pMMB208, Cm^R	This study
pMF800	*Pst*I/*Bam*HI PCR fragment of 5-prime FLAG™-*yscY* in pMMB208, Cm^R	This study
pJMG195	*Bam*HI/*Eco*RI PCR fragment of 5-prime FLAG™-*pscY* (*pcr4*)in pMMB208, Cm^R	This study
pJMG180	*Bam*HI/*Eco*RI PCR fragment of 5-prime FLAG™-*ascY* (*A. salmonicida*) in pMMB208, Cm^R	This study
pJMG181	*Bam*HI/*Eco*RI PCR fragment of 5-prime FLAG™-*ascY* (*A. hydrophila*) in pMMB208, Cm^R	This study
pJMG182	*Pst*I/*Bam*HI PCR fragment of 5-prime FLAG™-*vscY* (*V. harveyi*) in pMMB208, Cm^R	This study
pJMG183	*Bam*HI/*Eco*RI PCR fragment of 5-prime FLAG™-*vscY* (*V. parahaemolyticus*) in pMMB208, Cm^R	This study
pJMG184	*Bam*HI/*Eco*RI PCR fragment of 5-prime FLAG™-*sctY* (*P. luminescens*) in pMMB208, Cm^R	This study
pJMG267	*Bam*HI/*Eco*RI PCR fragment of codon-optimized 5-prime FLAG™-*pscY* (*P. aeruginosa*) in pMMB208, Cm^R	This study

pJMG268	*Bam*HI/*Eco*RI PCR fragment of codon-optimized 5-prime FLAG^{TM}-*ascY (A. salmonicida)* in pMMB208, Cm^R	This study
pJMG269	*Bam*HI/*Eco*RI PCR fragment of codon-optimized 5-prime FLAG^{TM}-*ascY (A. hydrophila)* in pMMB208, Cm^R	This study
pJMG270	*Pst*I/*Bam*HI PCR fragment of codon-optimized 5-prime FLAG^{TM}-*vscY (V. harveyi)* in pMMB208, Cm^R	This study
pJMG271	*Bam*HI/*Eco*RI PCR fragment of codon-optimized 5-prime FLAG^{TM}-*vscY (V. parahaemolyticus)* in pMMB208, Cm^R	This study
pJMG272	*Bam*HI/*Eco*RI PCR fragment of codon-optimized 5-prime FLAG^{TM}-*sctY (P. luminescens)* in pMMB208, Cm^R	This study
pGADT7	*LEU2,* Amp^R	Clontech Laboratories
pPJE026	*Eco*RI/*Bam*HI fragment of *yscX* in pGADT7, *LEU2,* Amp^R	(Bröms et al., 2005)
pPJE025	*Eco*RI/*Xho*I PCR fragment of *pscX* (*pcr3*) in pGADT7, *LEU2,* Amp^R	(Bröms et al., 2005)
pMF710	*Eco*RI/*Bam*HI PCR fragment of *vscX* (*V. parahaemolyticus*) in pGADT7, *LEU2,* Amp^R	This study
pMF711	*Eco*RI/*Bam*HI PCR fragment of *vscX* (*V. harveyi*) in pGADT7, *LEU2,* Amp^R	This study
pMF712	*Eco*RI/*Bam*HI PCR fragment of *sctX* in pGADT7, *LEU2,* Amp^R	This study
pMF717	*Eco*RI/*Bam*HI PCR fragment of *ascX* (*A. hydrophilia*) in pGADT7, *LEU2,* Amp^R	This study
pMF718	*Eco*RI/*Bam*HI PCR fragment of *ascX* (*A. salmonicida*) in pGADT7, *LEU2,* Amp^R	This study
pJMG043	*Eco*RI/*Xho*I PCR fragment of *yscV*(322-704) in pGADT7, *LEU2*, Amp^R	This study
pJMG217	*Nde*I/*Bam*HI PCR fragment of *pscV* (*P. aeruginosa*) in pGADT7, *LEU2*, Amp^R	This study
pJMG218	*Eco*RI/*Xho*I PCR fragment of *ascV* (*A. hydrophila*) in pGADT7, *LEU2,* Amp^R	This study
pJMG219	*Nde*I/*Bam*HI PCR fragment of *vscV* (*V. parahaemolyticus*) in pGADT7, *LEU2*, Amp^R	This study
pJMG220	*Eco*RI/*Xho*I PCR fragment of *sctV* (*P. luminescens*) in pGADT7, *LEU2,* Amp^R	This study
pGBKT7	*TRP1*, Km^R	Clontech Laboratories
pMF433	*Eco*RI/*Pst*I of *yscY* in pGBKT7, *TRP1*, Km^R	(Francis et al., 2001)
pPJE024	*Bam*HI/*Pst*I PCR fragment of *pscY* (*pcr4*) in pGBKT7, *TRP1*, Km^R	(Bröms et al., 2005)
pMF713	*Bam*HI/*Pst*I PCR fragment of *vscY* (*V. harveyi*) in pGBKT7, *TRP1*, Km^R	This study
pMF714	*Eco*RI/*Bam*HI PCR fragment of *ascY* (*A. salmonicida*) in pGBKT7, *TRP1*, Km^R	This study
pMF715	*Eco*RI/*Bam*HI PCR fragment of *vscY* (*V. parahaemolyticus*) in pGBKT7, *TRP1*, Km^R	This study
pMF716	*Eco*RI/*Bam*HI PCR fragment of *sctY* in pGBKT7, *TRP1*, Km^R	This study
pMF719	*Eco*RI/*Bam*HI PCR fragment of *ascY* (*A. hydrophilia*) in pGBKT7, *TRP1*, Km^R	This study
pBridge	*TRP1,* Amp^R	Clontech Laboratories
pSL114	360 bp *Eco*RI/*Pst*I PCR fragment of *yscY* in pGBT9, *TRP1*, Ap^R	(Francis et al., 2001)

pMF442	EcoRI/PstI fragment of full-length *yscY* (lifted from pSL114) in MCSI of pBridge, *TRP1,* Amp^R	This study
pJMG070	*Not*I/*Bgl*II PCR fragment of *yscX* in MCSII of pMF442 (pBridge derivative), *TRP1,* Amp^R	This study
pJMG213	pMF442; *Not*I/*Bgl*II PCR fragment of *pscX* in MCSII of pMF442 (pBridge derivative), *TRP1,* Amp^R	This study
pJMG214	pMF442; *Not*I/*Bgl*II PCR fragment of $ascX_{Ah}$ in MCSII of pMF442 (pBridge derivative), *TRP1,* Amp^R	This study
pJMG215	pMF442; *Not*I/*Bgl*II PCR fragment of $vscX_{Vp}$ in MCSII of pMF442 (pBridge derivative), *TRP1,* Amp^R	This study
pJMG216	pMF442; *Not*I/*Bgl*II PCR fragment of *sctX* in MCSII of pMF442 (pBridge derivative), *TRP1,* Amp^R	This study
pJMG208	*Eco*RI/*Pst*I PCR fragment of *pscY* in MCSI, and *Not*I/*Bgl*II PCR fragment of *pscX* in MCSII of pBridge, *TRP1,* Amp^R	This study
pJMG209	*Eco*RI/*Pst*I PCR fragment of $ascY_{Ah}$ in MCSI, and *Not*I/*Bgl*II PCR fragment of $ascX_{Ah}$ in MCSII of pBridge, *TRP1,* Amp^R	This study
pJMG210	*Eco*RI/*Pst*I PCR fragment of $vscY_{Vp}$ in MCSI, and *Not*I/*Bgl*II PCR fragment of $vscX_{Vp}$ in MCSII of pBridge, *TRP1,* Amp^R	This study
pJMG211	*Eco*RI/*Pst*I PCR fragment of *sctY* in MCSI, and *Not*I/*Bgl*II PCR fragment of *sctX* in MCSII of pBridge, *TRP1,* Amp^R	This study

Table S2| Oligonucleotides used in this study

Purpose/vector	Oligonucleotide name and sequence
**COMPLEMENTATION**	
pMF720 (*ascX*$_{As}$)	pascX(sa)-F, 5' – ACG *GAA TTC* GTT GCA GCG AGA GGT GC -3' (*Eco*RI) and pascX(sa)-R, 5' – ACG *GGA TCC* TCA TAC CTT GTG CAG C-3' (*Bam*HI)
pMF721 (*ascY*$_{As}$)	pascY(sa)-F, 5' – ACG *GAA TTC* GAC CCT TCA TCT GCT GC -3' (*Eco*RI) and pascY(sa)-R, 5' – ACG *GGA TCC* TCA AGG GTG CGT TGA TTC -3' (*Bam*HI)
pMF722 (*ascX*$_{Ah}$)	pMMBaX(hy)-F, 5' – ACG *GAA TTC* ATG ACA GCA AAC CGC AG -3' (*Eco*RI) and pascX(hy)-R, 5' – ACG *GGA TCC* TCA TAC CTT GTG CAG C -3' (*Bam*HI)
pMF723 (*ascY*$_{Ah}$)	pascY(hy)-F, 5' – ACG *GAA TTC* GAC CCT TCA TCT GCT GC -3' (*Eco*RI) and pascY(hy)-R, 5' – ACG *GGA TCC* TCA AGG GAA CGT TGA -3' (*Bam*HI)
pMF724 (*vscX*$_{vh}$)	pMMBvX(ha)-F, 5' – ACG *GAA TTC* GTT TAC ATA AAG ATG -3' (*Eco*RI) and pvscX(ha)-R, 5' – ACG *GGA TCC* TTA AAC CTG AAT CAG TAG -3' (*Bam*HI)
pMF796 (*vscY*$_{Ah}$)	pvscY(ha)-F, 5' – ACG T*GG ATC C*AA TCT ACT GAT TCA GG -3' (*Bam*HI) and pvscY(ha)-R, 5' – ACG *CTG CAG* TTA TTC ATT TGA TTC ACT A -3' (*Pst*I)
pMF725 (*vscX*$_{vp}$)	pMMBvX(pa)-F, 5' – ACG *GAA TTC* GCT TTT AGT GCG TCT TCA CG -3' (*Eco*RI) and pvscX(pa)-R, 5' – ACG *GGA TCC* TTA AAC CTG AAT AAG AAG -3' (*Bam*HI)
pMF726 (*vscY*$_{vp}$)	pvscY(pa)-F, 5' – ACG *GAA TTC* AAT CTT CTT ATT CAG G -3' (*Eco*RI) and pvscY(pa)-R, 5' – ACG *GGA TCC* TTA TTC ATG AGA TTC AC -3' (*Bam*HI)
pMF727 (*sctX*)	pMMBsX(lu)-F, 5' – ACG *GAA TTC* TTT GCT AAG AGT ACA GC -3' (*Eco*RI) and psctX(lu)-R, 5' – ACG *GGA TCC* TCA TAC CTT GTG CAA CAG G -3' (*Bam*HI)
pMF728 (*sctY*)	psctY(lu)-F, 5' – ACG *GAA TTC* GGC TCT GCA CCT GTT GCA -3' (*Eco*RI) and psctY(lu)-R, 5' – ACG *GGA TCC* ACG GTT CAT AAT AGA G -3' (*Bam*HI)
pMF733 (*ascXY*$_{As}$)	pascX(sa)-F and pascY(sa)-R
pMF734 (*ascXY*$_{Ah}$)	pMMB-aX(hy)-F and pascY(hy)-R
pMF735 (*vscXY*$_{vp}$)	pMMB-vX(pa)-F and pvscY(pa)-R
pMF736 (*sctXY*)	pMMB-sX(lu)-F and psctY(lu)-R
pMF797 (*vscXY*$_{vh}$)	pMMBvX(ha)-F, 5' – AC*G GAT CC*T CGT TTA CAT AAA GAT G-3' (*Bam*HI) and pvscY(ha)-R
pMF800 (FLAG™-*yscY*)	p(F)yscY, 5' –ACG T*CT GCA G*AA GGA GAT ATA CAT ATG GAC TAC AAG GAC GAC GAT GAC AAG ATG AAT ATT ACT TTA ACC- 3' (*Pst*I) and p(R)yscY, 5'- ACG T*GG ATC C*TC ATG GGG ATT CAT TAT G- 3' (*Bam*HI)
pJMG195 (FLAG™-*pscY*)	pFlagPscY-F, 5' –ACG T*GG ATC C*AA GGA GAT ATA CAT ATG GAC TAC AAG GAC GAC GAT GAC AAG ATG ACG TTG AAA CCG ACG- 3' (*Bam*HI) and pFlagPscY-R, 5' –GCA T*GA ATT C*TC ATT CCC GCG CCT CCA GC- 3' (*Eco*RI)
pJMG180 (FLAG™-*ascY*$_{As}$)	pFlagAscYAs-F, 5' –ACG T*GG ATC C*AA GGA GAT ATA CAT ATG GAC TAC AAG GAC GAC GAT GAC AAG ATG ACC ATG GTG CTT ACG- 3' (*Bam*HI) and pFlagAscYAs-R, 5' –GCA T*GA ATT C*TC AAG GGT GCG TTG ATT C- 3' (*Eco*RI)
pJMG181 (FLAG™-*ascY*$_{Ah}$)	pFlagAscYAh-F, 5' –ACG T*GG ATC C*AA GGA GAT ATA CAT ATG GAC TAC AAG GAC GAC GAT GAC AAG ATG ACC ATG GTT CTC TCG- 3' (*Bam*HI) and pFlagPscY-R, 5' –GCA T*GA ATT C*TC AAG GGA ACG TTG ATT C- 3' (*Eco*RI)
pJMG182 (FLAG™-*vscY*$_{Vh}$)	pFlagVscYVh-F, 5' –ACG T*CT GCA G*AA GGA GAT ATA CAT ATG GAC TAC AAG GAC GAC GAT GAC AAG ATG TTG CAA TCG AAA GAC G- 3' (*Pst*I) and pFlagVscYVh-R, 5' –GCA T*GG ATC C*TT ATT CAT TTG ATT CAC TA- 3' (*Bam*HI)
pJMG183 (FLAG™-*vscY*$_{Vp}$)	pFlagVscYVp-F, 5' –ACG T*GG ATC C*AA GGA GAT ATA CAT ATG GAC TAC AAG GAC GAC GAT GAC AAG ATG CTA AGC ACC AAA GAT ATT G- 3' (*Bam*HI) and pFlagVscYVp-R, 5' –GCA T*GA ATT C*TT ATT CAT GAG ATT CAC TA- 3' (*Eco*RI)
pJMG184 (FLAG™-*sctY*)	pFlagSctY-F, 5' –ACG T*GG ATC C*AA GGA GAT ATA CAT ATG GAC TAC AAG GAC GAC GAT GAC AAG ATG ACT CTC AGT GCT AAA CAG- 3' (*Bam*HI) and pFlagSctY-R, 5' –GCA T*GA ATT C*TC ATA ATA GAG CCC CTT G- 3' (*Eco*RI)

pJMG242 (FLAG™-*yscX*)	pFlagYscX-F, 5’-ACG T*GG ATC C*AA GGA GAT ATA CAT ATG GAC TAC AAG GAC GAC GAT GAC AAG GTG AGT CGC ATA ATA ACTG-3’ (*Bam*HI) and pHisYscX-R, 5’- GCAT *GAA TTC* TCA TAC TTT GTG CAA CAGG-3’ (*Eco*RI)
pJMG243 (FLAG™-*pscX*)	pFlagPscX-F, 5’-ACG T*GG ATC C*AA GGA GAT ATA CAT ATG GAC TAC AAG GAC GAC GAT GAC AAG ATG AGC CGG GTC GGT GCC TGGC-3’ (*Bam*HI) and pHisPscX-R
pJMG244 (FLAG™-*ascX*$_{As}$)	pFlagAscX(As)-F, 5’-ACG T*GG ATC C*AA GGA GAT ATA CAT ATG GAC TAC AAG GAC GAC GAT GAC AAG ATG AGC CGG ATC ACT GCCG-3’ (*Bam*HI) and pHisAscXAs-R
pJMG245 (FLAG™-*ascX*$_{Ah}$)	pFlagAscX(Ah)-F, 5’-ACG T*GG ATC C*AA GGA GAT ATA CAT ATG GAC TAC AAG GAC GAC GAT GAC AAG ATG AGT CGA ATT ACT GCTG-3’ (*Bam*HI) and pHisAscXAs-R
pJMG246 (FLAG™-*vscX*$_{Vh}$)	pFlagVscX(Vh)-F, 5’-ACG T*GG ATC C*AA GGA GAT ATA CAT ATG GAC TAC AAG GAC GAC GAT GAC AAG ATG AGT CGC ATT AGT ACG-3’ and pHisVscXVh-R
pJMG247 (FLAG™-*vscX*$_{Vp}$)	pFlagVscX(Vp)-F, 5’-ACG T*GG ATC C*AA GGA GAT ATA CAT ATG GAC TAC AAG GAC GAC GAT GAC AAG TTG ATG ACC AGA GTT AGT AC-3’ and pHisVscXVp-R
pJMG248 (FLAG™-*sctX*)	pFlagSctX-F, 5’-ACG T*GG ATC C*AA GGA GAT ATA CAT ATG GAC TAC AAG GAC GAC GAT GAC AAG GTG AGC AAA ATC GCT GCCG-3’ and pHisSctX-R
**YEAST TWO HYBRID**	
pMF710 (*vscX*$_{Vp}$)	pvscX(pa)-F, 5’ – ACG *GAA TTC* TTG ATG ACC AGA GTT AG -3’ (*Eco*RI) and pvscX(pa)-R
pMF711 (*vscX*$_{Vh}$)	pvscX(ha)-F, 5’ – ACG *GAA TTC* ATG AGT CGC ATT AGT ACG -3’ (*Eco*RI) and pvscX(ha)-R
pMF712 (*sctX*)	psctX(lu)-F, 5’ – ACG *GAA TTC* GTG AGC AAA ATC GCT GCC G -3’ (*Eco*RI) and psctX(lu)-R
pMF717 (*ascX*$_{Ah}$)	pascX(hy)-F, 5’ – ACG *GAA TTC* ATG AGT CGA ATT ACT G -3’ (*Eco*RI) and pascX(hy)-R
pMF718 (*ascX*$_{As}$)	pascX(sa)-F, 5’ – ACG *GAA TTC* ATG AGC CGG ATC ACT GCC -3’ (*Eco*RI) and pascX(sa)-R
pMF713 (*vscY*$_{vh}$)	pvscY(ha)-F, 5’ – AC*G GAT CC*C CAT GTT GCA ATC GAA AGA CG -3’ (*Bam*HI) and pvscY(ha)-R
pMF714 (*ascY*$_{As}$)	pascY(sa)-F, 5’ – ACG *GAA TTC* ATG ACC ATG GTG CTT ACG -3’ (*Eco*RI) and pascY(sa)-R
pMF715 (*vscY*$_{Vp}$)	pvscY(pa)-F, 5’ – ACG *GAA TTC* ATG CTA AGC ACC AAA GAT -3’ (*Eco*RI) and pvscY(pa)-R
pMF716 (*sctY*)	psctY(lu)-F, 5’ – ACG *GAA TTC* ATG ACT CTC AGT GCT -3’ (*Eco*RI) and psctY(lu)-R
pMF719 (*ascY*$_{Ah}$)	pascY(hy), 5’- ACG *GAA TTC* ATG ACC ATG GTT CTC TCG -3’ (*Eco*RI) and pascY(hy)-R
**YEAST THREE HYBRID** (cloning into pBridge MCSI and MCSII)	
pJMG070 (a pMF442 derivative with *yscX* in MCSII)	pMFYscX-F, 5’ –GCA TGT *GCG GCC GC*C GTG AGT CGC ATA ATA -3’ (*Not*I) and pMFYscX-R, 5’ -GCA TGT *AGA TCT* TCA TAC TTT GTG CAA CAG -3’ (*Bgl*II)
PJMG213 (a pMF442 derivative with *pscX* in MCSII)	pBriPscX-F, 5’-GCA TAT *GCG GCC GC*T ATG AGC CGG GTC GGT G -3’ (*Not*I) and pBriPscX-R, 5’-GCA TGT *AGA TCT* TCA TAC CTT GTG CAA CAG -3’ (*Bgl*II)
pJMG214 (a pMF442 derivative with *ascX*$_{Ah}$ in MCSII)	pBriAscX(Ah)-F, 5’-GCA TAT *GCG GCC GC*C ATG AGT CGA ATT ACT GC -3’ (*Not*I) and pBriAscX(Ah)-R, 5’-GCA TGT *AGA TCT* TCA TAC CTT GTG CAG CAG-3’ (*Bgl*II)
pJMG215 (a pMF442 derivative with *vscX*$_{Vp}$ in MCSII)	pBriVscX(Vp)-F, 5’-GCA TAT *GCG GCC GC*C TTG ATG ACC AGA GTT AG -3’ (*Not*I) and pBriVscX(Vp)-R, 5’-GCA TGT *AGA TCT* TTA AAC CTG AAT AAG AAG-3’ (*Bgl*II)
pJMG216 (a pMF442 derivative with *sctX* in MCSII)	pBriSctX-F, 5’-GCA TAT *GCG GCC GC*C GTG AGC AAA ATC GCT G-3’ (*Not*I) and pBriSctX-R, 5’-GCA TGT *AGA TCT* TCA TAC CTT GTG CAA CAG GTG-3’ (*Bgl*II)
pJMG208 (*pscY, pscX*)	pBriPscY-F, 5’- ACGT *GAA TTC* ATG ACG TTG AAA CCG ACG -3’ (*Eco*RI) and

	pBriPscY-R, 5’- ACGT *CTG CAG* TCA TTC CCG CGC CTC CAG -3’ (*Pst*I)<br>pBriPscX-F and pBriPscX-R
pJMG209 (*ascYAh*, $ascX_{Ah}$)	pBriAscY(Ah)-F, 5’- ACGT *GAA TTC* ATG ACC ATG GTT CTC TCG -3’ (*Eco*RI) and pBriAscY(Ah)-R, 5’- ACGT *CTG CAG* TCA AGG GAA CGT TGA TTC -3’ (*Pst*I)<br>pBriAscX(Ah)-F and pBriAscX(Ah)-R
pJMG210 ($vscY_{Vp}$, $vscX_{Vp}$)	pBriVscY(Vp)-F, 5’- ACGT *GAA TTC* ATG CTA AGC ACC AAA GAT ATTG -3’ (*Eco*RI) and pBriVscY(Vp)-R, 5’- ACGT *CTG CAG* TTA TTC ATG AGA TTC ACT ATG -3’ (*Pst*I)<br>pBriVscX(Vp)-F and pBriVscX(Vp)-R
pJMG211 (*sctY, sctX*)	pBriSctY-F, 5’- ACGT *GAA TTC* ATG ACT CTC AGT GCT AAA CAG -3’ (*Eco*RI) and pBriSctY-R, 5’- ACGT *CTG CAG* TCA TAA TAG AGC CCC TTG -3’ (*Pst*I)<br>pBriSctX-F and pBriSctX-R
pJMG043 (*yscV*)	pY2HyscV322-704(Ec), 5’- ACG GAA TTC GTA AGC AGA AGT CGT AAT G -3’ (*Eco*RI) and pY2HyscV322-704(Xh), 5’- ACG CTC GAG TCA TAA GCA AAT TCG TCC AAG -3’ (*Xho*I)
pJMG217 (*pscV*)	pGADPscV(322-706)-F, 5’- ATAT *CAT ATG* CGC CAG CGG GCC CAG GCC AG -3’ (*Nde*I) and pGADPscV(322-706)-R, 5’- ACGT *GGA TCC* TCA CAA CAC GAT CCT GCC AAG -3’ (*Bam*HI)
pJMG218 ($ascV_{Ah}$)	pGADAscVAh(339-721)-F, 5’- ACGT *GAA TTC* CGT CAG CGT AAG GAG CAG -3’ (*Eco*RI) and pGADAscVAh(339-721)-R, 5’- ACGT *CTC GAG* TCA CAG GCA GAC CCT CCC GAG -3’ (*Xho*I)
pJMG219 ($vscV_{Vp}$)	pGADVscV(325-708)-F, 5’- ACGT *CAT ATG* CAA CAG AAA AAG CAA ACC G -3’ (*Nde*I) and pGADVscV(325-708)-R, 5’- ACGT *GGA TCC* TTA CAT CCC TAC CCG TCC AAG -3’ (*Bam*HI)
pJMG220 (*sctV*)	pGADSctV(321-705)-F, 5’- ACGT *GAA TTC* CAG CGT CAG CGC CAA GCT AAC -3’ (*Eco*RI) and pGADSctV(321-705)-R, 5’- ACGT *CTC GAG* TCA TAG ACA CAC TCT ACC AAG -3’ (*Xho*I)
**QUALITATIVE RT-PCR**	
rpoA (~330 bp)	prpoAa, 5’ - GTT CGA CGC ACG CCA AGG TGA -3’ and prpoAb, 5’ - ACG TCC TGC GGC TTG ACG AT – 3’
yscX (~317 bp)	pRTYscX-F, 5’ - TGT CGG CGA TTA GCC TGG AAG -3’ and pRTYscX-R, 5’ - GTG CAA CAG GTT AAG GAT TA -3’
pscX (~313 bp)	pRTPscXopt-F, 5’ - CAC ATT GGG ATT GAA CGC TTAG -3’ and pRTPscXopt-R, 5’ - GTA ACA GAC GCT CAT CCTC -3’
$ascX_{As}$ (~333 bp)	pRTAscXAsopt-F, 5’ - AGC ACA CAT TGG GAT TGA ACAG -3’ and pRTAscXAsopt-R, 5’ - AGG TGT AAG GTC ATC TGC -3’
$ascX_{Ah}$ (~329 bp)	pRTAscXAhopt-F, 5’ - CAC ATT GGT ATT GAA CAG TTG AG -3’ and pRTAscXAhopt-R, 5’ - AGG TGT AAG GTC ATT TGC -3’
$vscX_{Vh}$ (~338 bp)	pRTVscXVhopt-F, 5’ - AGC ACG TTG AAT GTG GGT ATTG -3’ and pRTVscXVhopt-R, 5’ - ATC GTC AGA TAG CGT TGA TCC AG -3’
$vscX_{Vp}$ (~351 bp)	pRTVscXVpopt-F, 5’ - AGC ACC CTG AAT GTT GGT ATTG – 3’ and pRTVscXVpopt-R, 5’ - AAC AGG TTCA ACG CCA TCG TC -3’
sctX (~310 bp)	pRTSctXopt-F, 5’ - AAC AGT TGA CCG CCA TTA GC -3’ and pRTSctXopt-F, 5’ - ACG CCA TCT GTA ATA ACT GC -3’
yscY (~315 bp)	pRTYscY-F, 5’ - CGA CAA CAG GAG TTC TTG CTG -3’ and pRTYscY-R, 5’ - TTC ATT ATG ATC TTT CAA CTC -3’
pscY (~309 bp)	pRTPscY-F, 5’ - TGA AAC CTA CGC AAC AACG -3’ and pRTPscY-R, 5’ - CTA ACT CCA AGG CAC GAGC -3’
$ascY_{As}$ (~339 bp)	pRTAscYAsopt-F, 5’ - ACG ATG GTT CTG ACC TCAC -3’ and pRTAscYAsopt-R, 5’ - GTT GAC TCG TTC TGC TCT TC -3’
$ascY_{Ah}$ (~295 bp)	pRTAscYAhopt-F, 5’ - GAC GGG TTG GTT ACA GTT AC -3’ and pRTAscYAhopt-R, 5’ - TGC TCT CGT TCT GCT CCTC -3’

vscY$_{Vh}$ (~314 bp)	pRTVscYVhopt-F, 5’ - ACA AAG CAA AGA TGT GGAG -3’ and pRTVscYVhopt-R, 5’ - TCA GGC TTT GCA GAT AAC GAC -3’
vscY$_{Vp}$ (~306 bp)	pRTVscYVpopt-F, 5’ - AGT TCA TGC AGC GTT ACA AG -3’ and pRTVscYVpopt-R, 5’ - TGG CTC TCG CTG TGT AAGG -3’
sctY (~262 bp)	pRTSctYopt-F, 5’ - ACA GTT ACA GTA TGG TCA CCC AG -3’ and pRTSctYopt-R, 5’ - AGC CAG ATA ACG CTG ATA CG -3’

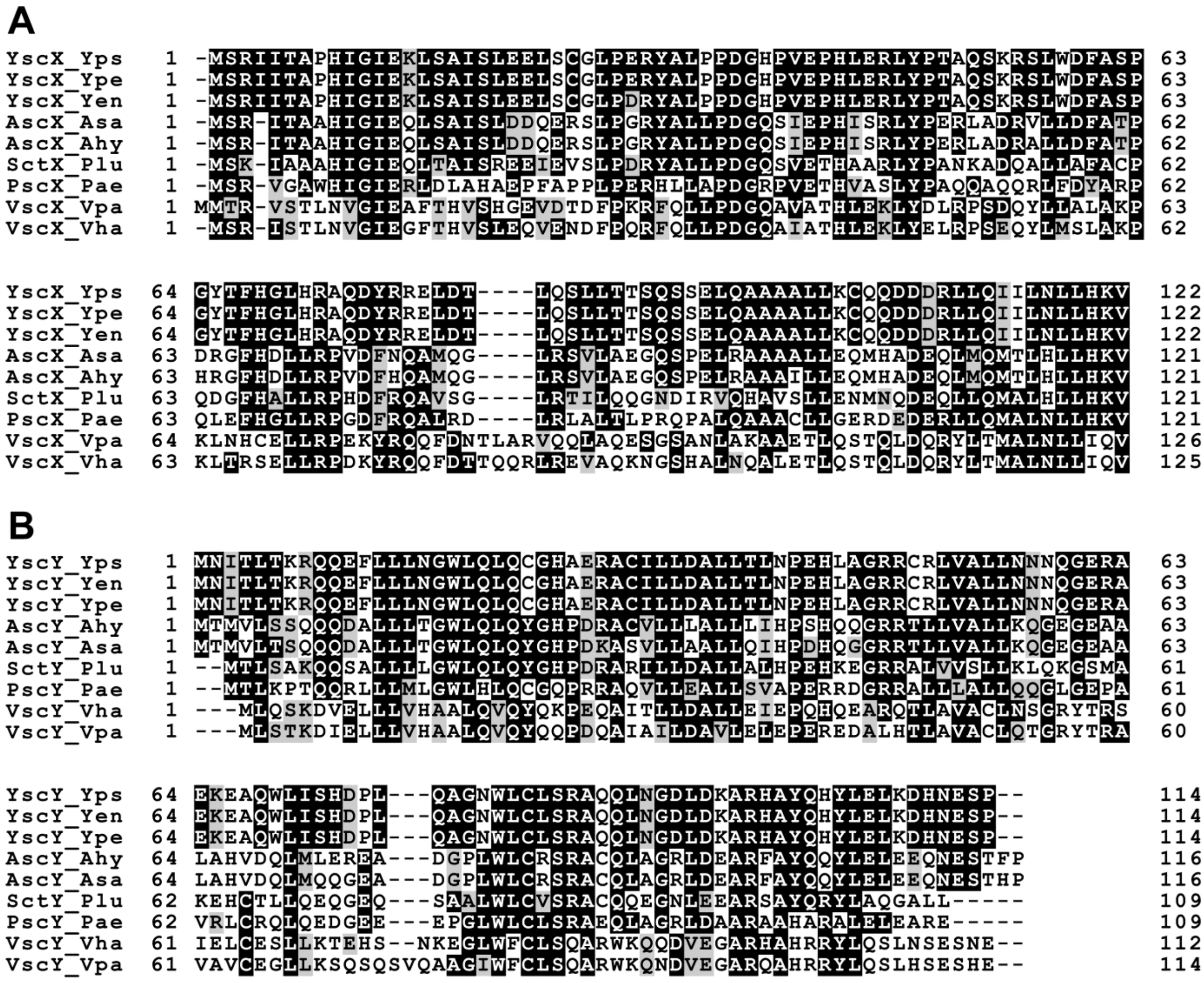

Figure S1| Amino acid sequence alignment among YscX and YscY protein families. YscX and YscY query sequences from *Y. pseudotuberculosis* designated YscX_Yps (GI number, 51593904) (**A**) or YscY_Yps (GI number, 51593903) (**B**) respectively were used to retrieve related alleles from NCBI genome database. This search identified sequences from representative bacteria with the following GI reference numbers in parentheses; Ype*, Yersinia pestis* (YscX, 16082724 and YscY, 16082723); Yen*, Yersinia enterocolitica* (YscX, 4324343 and YscY, 4324342); Asa*, Aeromonas salmonicida* (AscX, 66947966 and AscY, 66947967); Ahy*, Aeromonas hydrophilia* (AscX, 46398260 and AscY, 46398261); Pae*, Pseudomonas aeruginosa* (PscX, 62865832 and PscY, 62865833); Plu*, Photorhabdus luminescens* (SctX, 36787060 and SctY, 36787059); Vha*, Vibrio harveyi* (VscX, 41834176 and VscY, 41834175); Vha*, Vibrio parahaemolyticus* (VscX, 28898438 and VscY, 28898437). Sequences were then aligned using ClustalW (http://www.ebi.ac.uk/Tools/clustalw/index.html). Identical and similar amino acids are shaded grey and black respectively.

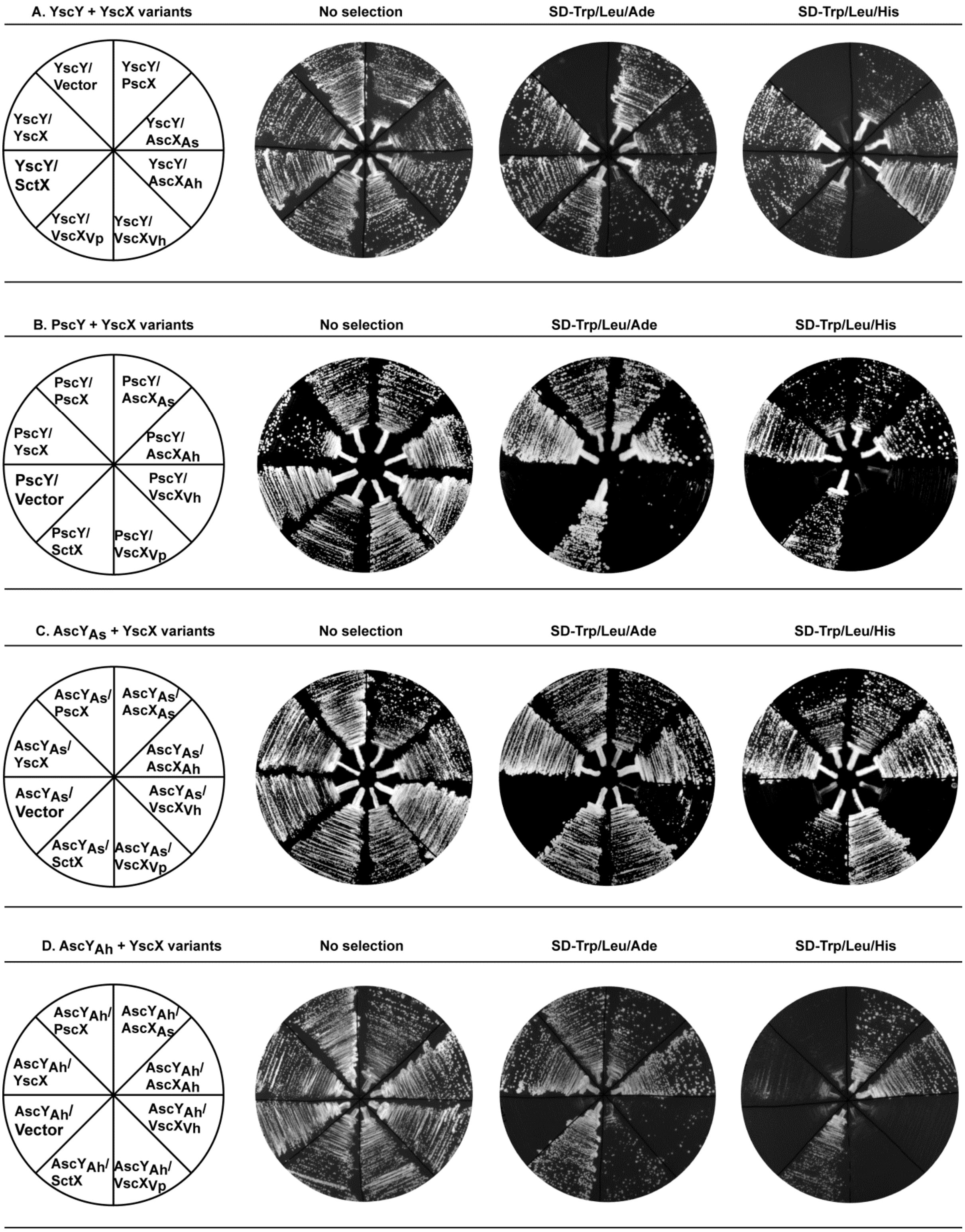

A. YscY + YscX variants
No selection
SD-Trp/Leu/Ade
SD-Trp/Leu/His
YscY/ Vector
YscY/ PscX
YscY/ YscX
YscY/ AscXAs
YscY/ SctX
YscY/ AscXAh
YscY/ VscXVp
YscY/ VscXVh
B. PscY + YscX variants
No selection
SD-Trp/Leu/Ade
SD-Trp/Leu/His
PscY/ PscX
PscY/ AscXAs
PscY/ YscX
PscY/ AscXAh
PscY/ Vector
PscY/ VscXVh
PscY/ SctX
PscY/ VscXVp
C. AscYAs + YscX variants
No selection
SD-Trp/Leu/Ade
SD-Trp/Leu/His
AscYAs/ PscX
AscYAs/ AscXAs
AscYAs/ YscX
AscYAs/ AscXAh
AscYAs/ Vector
AscYAs/ VscXVh
AscYAs/ SctX
AscYAs/ VscXVp
D. AscYAh + YscX variants
No selection
SD-Trp/Leu/Ade
SD-Trp/Leu/His
AscYAh/ PscX
AscYAh/ AscXAs
AscYAh/ YscX
AscYAh/ AscXAh
AscYAh/ Vector
AscYAh/ VscXVh
AscYAh/ SctX
AscYAh/ VscXVp

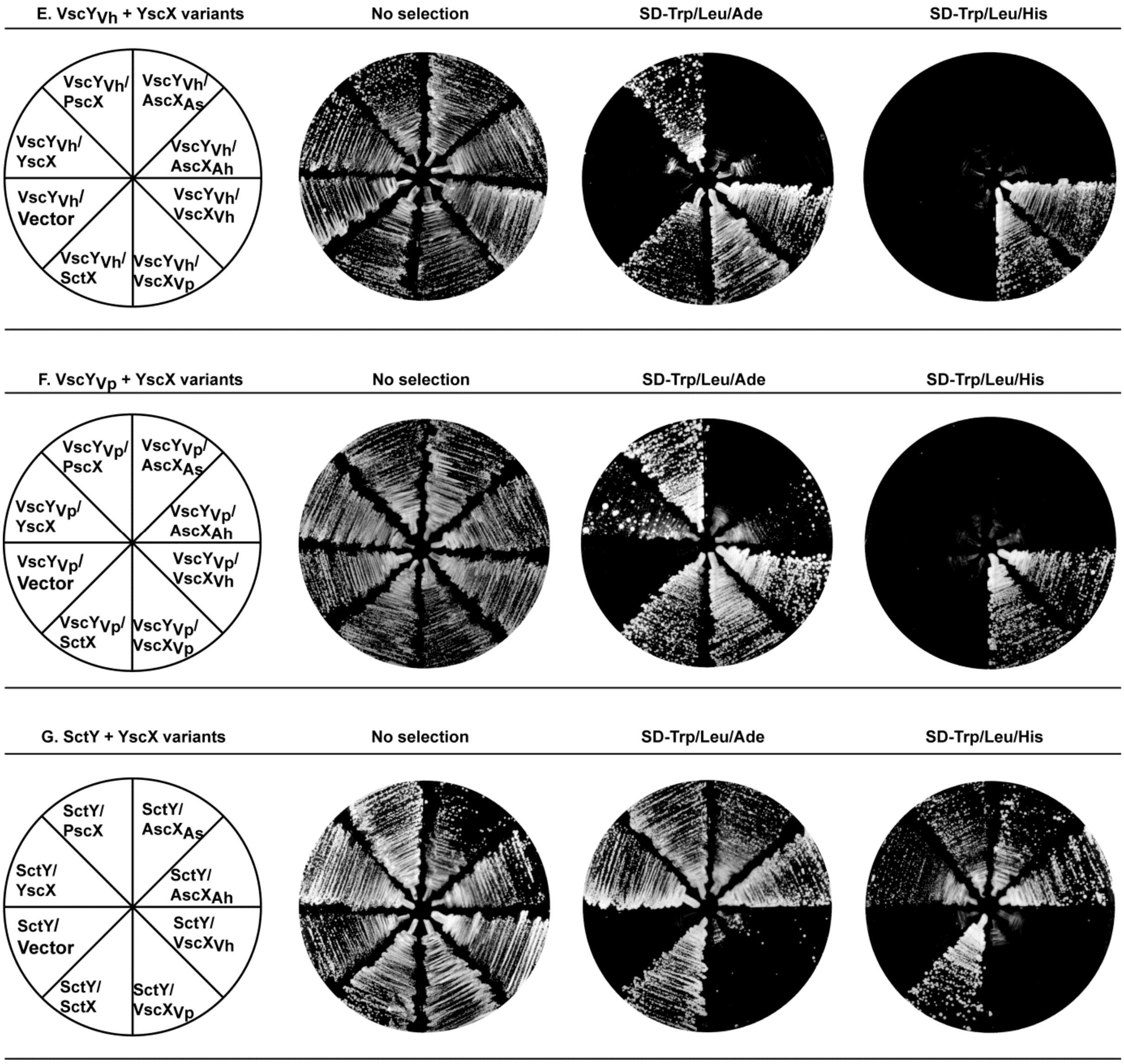

Figure S2| Reciprocal interactions between YscX family members and YscY family members generated in the yeast two-hybrid assay. All *yscX*-like alleles were established as fusions to the GAL4 activation domain in the pGADT7 plasmid. All *yscY*-like alleles were established as fusions to the GAL4 DNA binding domain in the pGBKT7 plasmid. Pairwise combinations of pGADT7- and pGBKT7-derivatives were co-transformed into *S. cerevesiae* AH109 and maintained by growing on a SD minimal media deprived of tryptophan and leucine (No selection). A positive interaction between the YscX-like and YscY-like fusions was detected through activation of either the *HIS3* reporter gene or the *ADE2* reporter gene following growth of AH109 yeast cells on selective SD minimal media lacking tryptophan, leucine and adenine (SD-Trp/Leu/Ade) or histidine (SD-Trp/Leu/His). The intrinsic leakiness of *HIS3* reporter gene was neutralized by supplementation of 4 mM 3-aminotriazole to the histidine minus SD minimal media. The schematic template on the far left panel represents the different combinations tested (**A to G**). An interaction was defined as strong if yeast growth on selective plates was robust, intermediate if the growth was more modest and none if there was no growth. The result shown is a representative of three independent experiments.

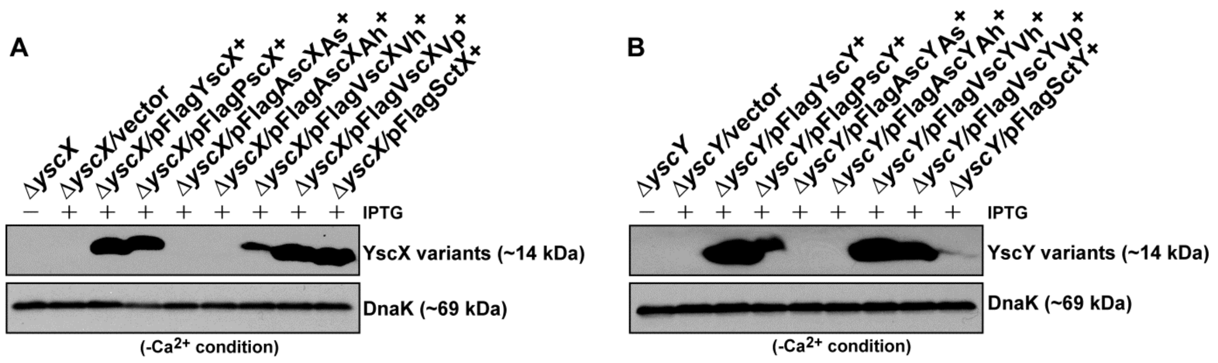

Figure S3| Protein expression profiles of native YscX and YscY and their homologues in *Y. pseudotuberculosis*. Bacterial strains were grown in secretion permissive BHI minus Ca^{2+} media. Protein samples associated with bacterial pellet were normalized to bacterial cell number and fractionated on 15% SDS-PAGE (YscX/YscY) or 12% SDS-PAGE (DnaK). Monoclonal anti-FLAG™ antiserum was used to detect the synthesized YscX protein family (**A**) and YscY protein family (**B**). Polyclonal anti-DnaK antiserum was used to detect the cytoplasmic molecular chaperone DnaK, and this served as a loading control. The '–' symbol indicates bacterial growth in the absence of IPTG, while the '+' symbol indicates that a final concentration of 0.4 mM IPTG was added in the culture media. The result shown is a representative of three independent experiments. Molecular mass values shown in parentheses were deduced from primary amino acid sequences.

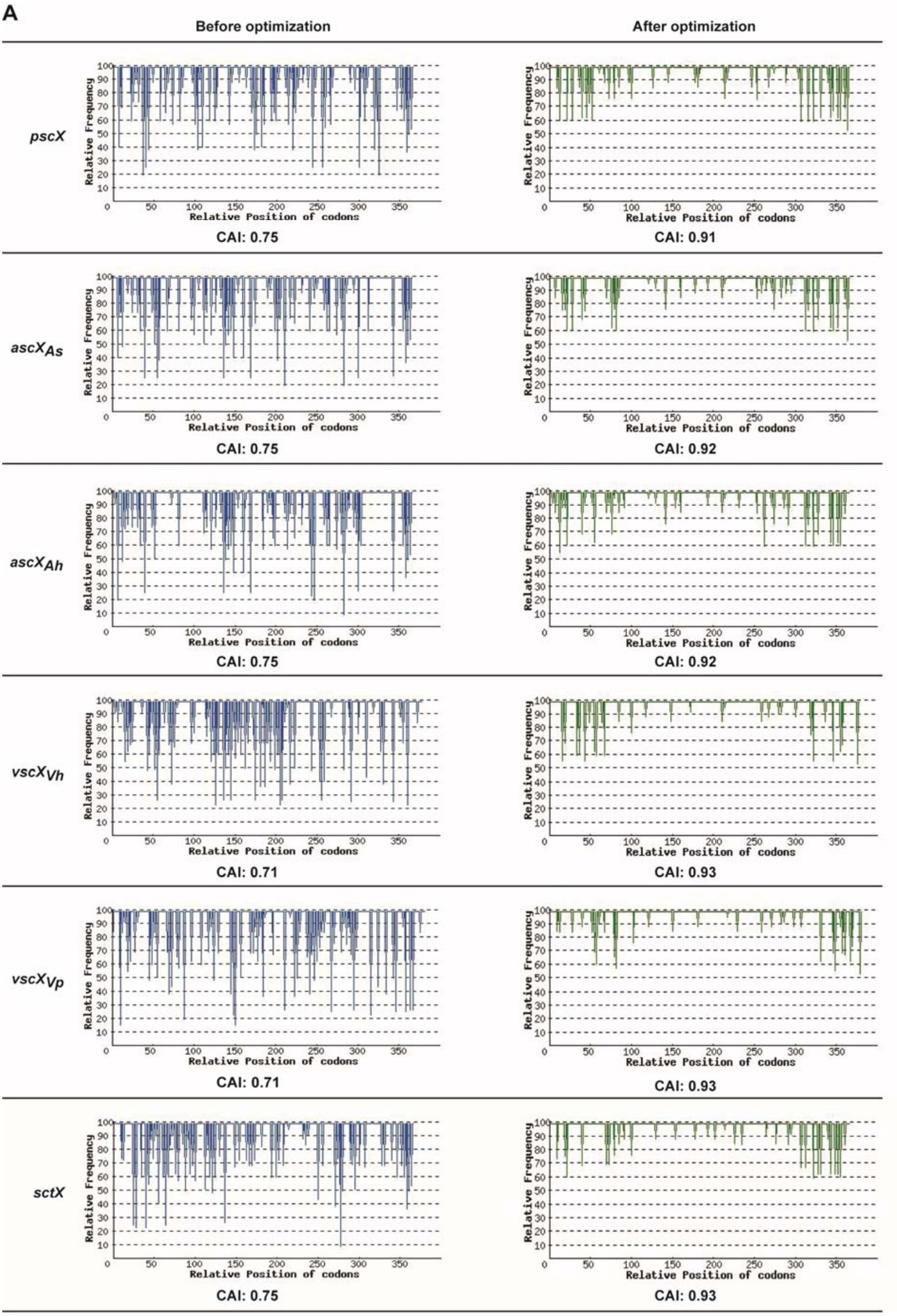
A
Before optimization
After optimization
pscX
CAI: 0.75
CAI: 0.91
ascX$_{As}$
CAI: 0.75
CAI: 0.92
ascX$_{Ah}$
CAI: 0.75
CAI: 0.92
vscX$_{Vh}$
CAI: 0.71
CAI: 0.93
vscX$_{Vp}$
CAI: 0.71
CAI: 0.93
sctX
CAI: 0.75
CAI: 0.93
Relative Frequency
Relative Position of codons

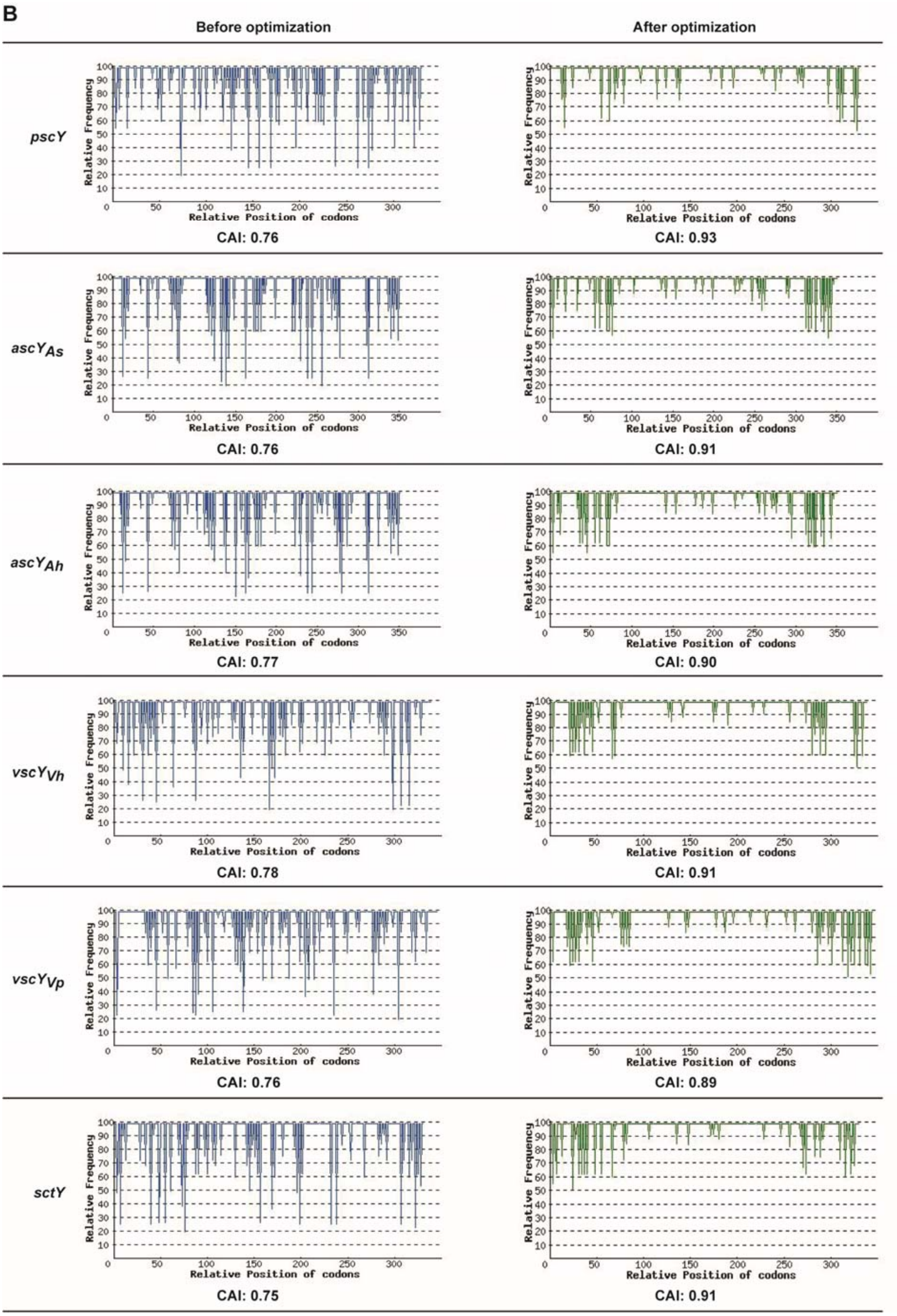
B
Before optimization
After optimization
pscY
$ascY_{As}$
$ascY_{Ah}$
$vscY_{Vh}$
$vscY_{Vp}$
sctY
Relative Frequency
Relative Position of codons
CAI: 0.76
CAI: 0.93
CAI: 0.76
CAI: 0.91
CAI: 0.77
CAI: 0.90
CAI: 0.78
CAI: 0.91
CAI: 0.76
CAI: 0.89
CAI: 0.75
CAI: 0.91

Figure S4| Codon-usage bias adjustment of *yscX*-like alleles and *yscY*-like alleles. A series of *yscX*-like and *yscY*-like genes were commercially synthesized by Genscript USA Inc. (Piscataway, NJ, USA) as codon-optimized variants that still encoded for the exact same amino acid sequence as the native gene. Codon adaptive index (CAI) was used to measure the codon usage bias for each codon in all *yscX*-like (**A**) and *yscY*-like genes (**B**). CAI is the ratio of usage of each codon to that of most abundant codon for the same amino acid in the genome of desired expression organism and assigns a value between 0 and 1.0. A CAI score of 1.0 is considered to be the preferred codon usage in the desired expression organism, while a CAI value of > 0.8 is considered to be good. By utilizing this information, we then adjusted the codon usage bias in *Y. pseudotuberculosis* by upgrading the CAI value as indicated at the bottom of each panel. In addition, the plots also provide the relative frequency of codon usage along the gene length sequence. The codon score is highest when the frequency of preferentially used codon is highest.

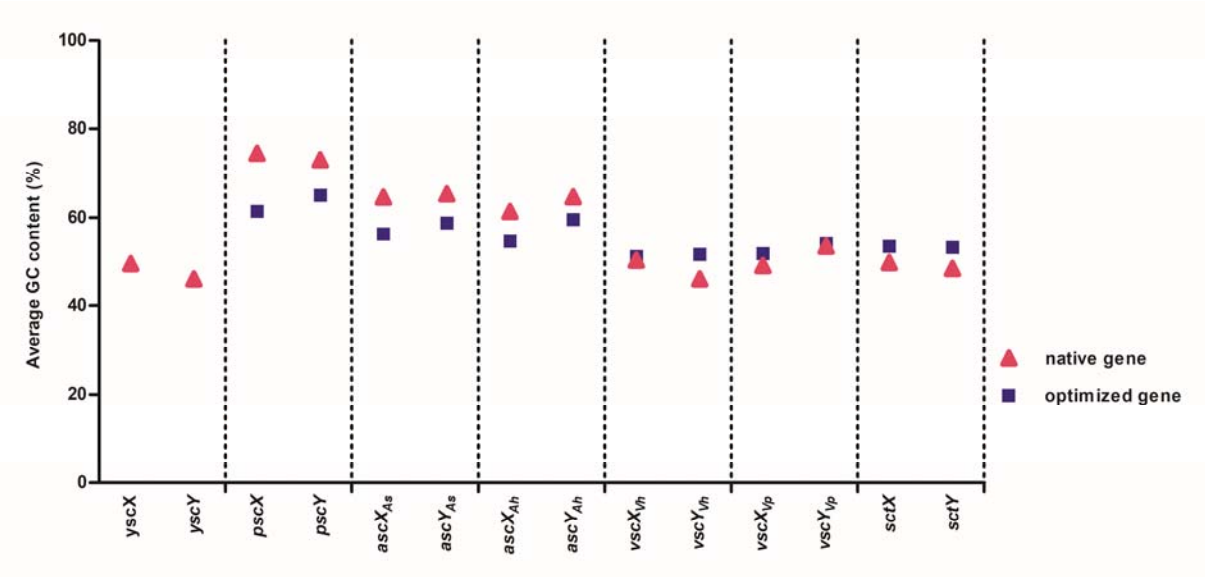

Figure S5| Comparison of the GC content between native and codon-optimized *yscX*-like alleles and *yscY*-like alleles. The average GC% was compared between the native (red triangle) gene and its GC content optimized variants (blue square) but both encoding the same amino acids. The average GC content in the nucleotide sequences of *yscX* and *yscY* from *Yersinia* was ~49.60 and 46.10 respectively. The percent GC content of native sequences of *yscX* and *yscY* homologues were highly distributed; ~73 for *P. aeruginosa* genes (*psc*), ~64 for *A. salmonicida* genes (asc_{As}), ~62 for *A. hydrophila* genes (asc_{Ah}), ~48 for *V. harveyi* genes (vsc_{Vh}), ~51 for *V. parahaemolyticus* genes (vsc_{Vp}) and ~49 for *P. luminescens* genes (*sct*). Following reconstitution of gene by GC optimization, the GC content was ~55% comparable to the GC content of *Yersinia* genome.

A

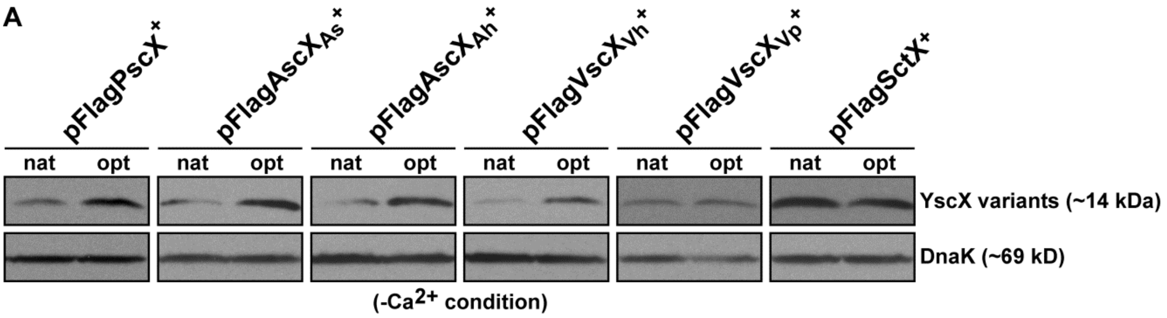

B

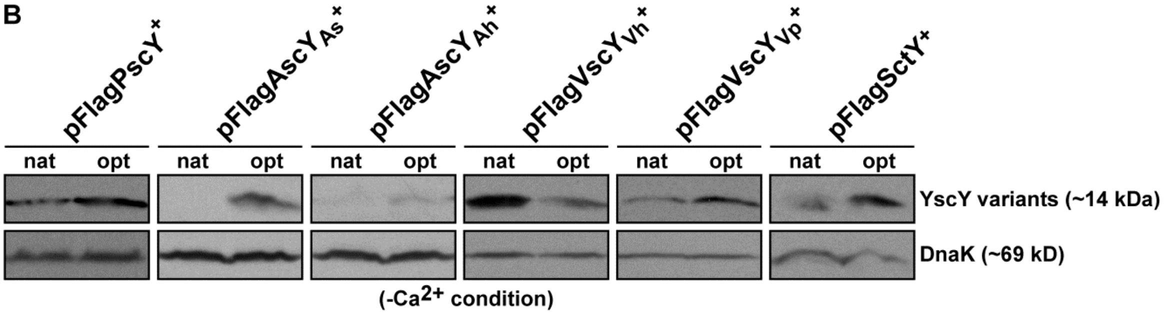

C

Fold change in accumulated levels of optimized YscX variants

YscX variant	Ratio optimised vs native (mean ± SD)
PscX	2.59±1.52
$AscX_{As}$	4.25±1.19
$AscX_{Ah}$	5.72±3.53
$VscX_{Vh}$	7.07±5.86
$VscX_{Vp}$	1.31±0.37
SctX	0.74±0.20

D

Fold change in accumulated levels of optimized YscY variants

YscY variant	Ratio optimized vs native (mean ± SD)
PscY	1.38±0.28
$AscY_{As}$	nd
$AscY_{Ah}$	nd
$VscY_{Vh}$	0.56±0.16
$VscY_{Vp}$	2.98±1.73
SctY	2.18±0.65

Figure S6| Relative protein levels of accumulated native and optimized YscX and YscY homologues in *Y. pseudotuberculosis*. The expression of both FLAG™-tagged native (nat) as well as optimized (opt) YscX (in the Δ*yscX* background) and YscY (Δ*yscY* background) variants was induced in strains by the addition of IPTG during growth in secretion permissive BHI minus Ca^{2+} medium. Proteins associated with the bacterial pellets were separated by 15% SDS-PAGE and analyzed by western blotting. Monoclonal anti- FLAG™ antiserum was used to detect the YscX variants (**A**) and YscY variants (**B**). Detection of DnaK with polyclonal anti-DnaK antiserum served as a loading control. Relative levels of accumulated FLAG™-tagged proteins were quantified from specific protein bands appearing on X-ray film after western blot detection, and following normalization to the corresponding loading control. The fold change of these relative levels as a result of codon optimization was determined by calculating the ratio between accumulated optimized and native YscX variants (**C**) and YscY variants (**D**). For two of the YscY homologues, $AscY_{As}$ and $AscY_{Ah}$, the level of accumulated native protein was too low to give a clear band on the X-ray film. Despite consistent success in detection of the optimized versions of these two YscY homologues, the fold change of

accumulated protein could therefore not be determined (nd = not determined). The results shown are representative of at least three independent experiments.

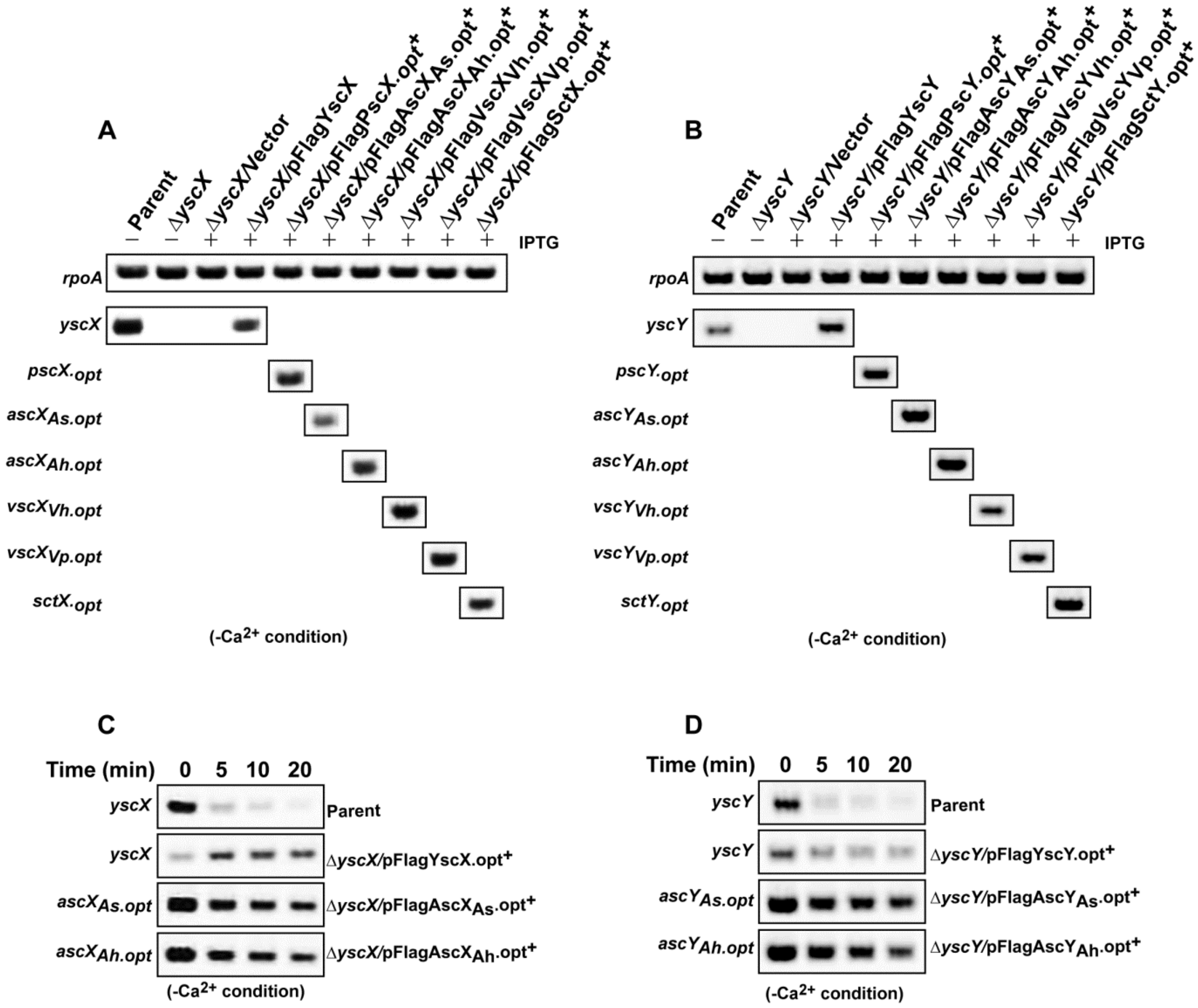

Figure S7| Analysis of gene transcription by qualitative RT-PCR. Total RNA was isolated from log-phase *Yersinia* cultures grown at 37° C in secretion permissive condition (BHI minus Ca^{2+}). Following reverse transcription, presence of *yscX*-like transcript (**A**) and *yscY*-like transcript (**B**) was determined by PCR using primers specific for the genes indicated at the side of each panel. RT-PCR analysis of *rpoA* was used as an internal control. IPTG to a final concentration of 0.4 mM was added where indicated to induce ectopic expression. Images were first visualized using a Chemidoc XRS (Bio-Rad) and acquired using the Quantity One 1-D software (Bio-Rad). Strains (**A**): Parent (YPIII/pIB102); Δ*yscX* (YPIII/pIB880); complemented YPIII/pIB880, pMMB208 (Vector); complemented YPIII/pIB880, pJMG242 (FLAG™-YscX^{+}); complemented YPIII/pIB880, pJMG261(FLAG™-PscX$_{.opt}^{+}$); complemented YPIII/pIB880, pJMG262 (FLAG™-AscX$_{As.opt}^{+}$); complemented YPIII/pIB880, pJMG263 (FLAG™-AscX$_{Ah.opt}^{+}$); complemented YPIII/pIB880, pJMG264 (FLAG™-VscX$_{Vh.opt}^{+}$); complemented YPIII/pIB880, pJMG265 (FLAG™-VscX$_{Vp.opt}^{+}$); complemented YPIII/pIB880, pMF266 (FLAG™-SctX$_{.opt}^{+}$). Strains (**B**): Parent (YPIII/pIB102); Δ*yscY* (YPIII/pIB890); complemented YPIII/pIB890, pMF800 (FLAG™-YscY+); complemented YPIII/pIB890, pJMG267 (FLAG™-PscY.opt+); complemented YPIII/pIB890, pJMG268 (FLAG™-AscY$_{As.opt}^{+}$); complemented YPIII/pIB890, pJMG269 (FLAG™-AscY$_{Ah.opt}^{+}$); complemented YPIII/pIB890, pJMG270 (FLAG™-VscY$_{Vh.opt}^{+}$); complemented YPIII/pIB890, pJMG271 (FLAG™-VscY$_{Vp.opt}^{+}$); complemented YPIII/pIB890, pJMG272 (FLAG™-SctY$_{.opt}^{+}$).

Representative *Yersinia* strains were used to measure the stability of *yscX*-like mRNA (**C**) and/or *yscY*-like mRNA (**D**) by using qualitative RT-PCR. *Yersiniae* cultures were grown in BHI minus Ca^{2+} as described above. After removing one volume of bacterial samples at time point zero, rifampicin (100 μg/ml) was added to the *Yersiniae* cultures to suppress RNA synthesis. Additional samples were then taken out at indicated time points (5, 10 and 20 min). Following reverse transcription, stability of mRNA was determined by PCR using specific primers for genes indicated at the side of each panel. Strains (C): Parent (YPIII/pIB102); complemented YPIII/pIB880, pJMG242 (FLAG™-YscX^{+}); complemented YPIII/pIB880, pJMG262 (FLAG™-AscX$_{As.opt}^{+}$); complemented YPIII/pIB880, pJMG263 (FLAG™-AscX$_{Ah.opt}^{+}$). Strains (D): Parent (YPIII/pIB102); complemented YPIII/pIB890, pMF800 (FLAG™-YscY+); complemented YPIII/pIB890, pJMG268 (FLAG™-AscY$_{As.opt}^{+}$); complemented YPIII/pIB890, pJMG269 (FLAG™-AscY$_{Ah.opt}^{+}$).

REFERENCES

Bölin, I., and Wolf-Watz, H. (1984). Molecular cloning of the temperature-inducible outer membrane protein 1 of Yersinia pseudotuberculosis. *Infect Immun* 43**,** 72-78.

Bradley, D.E. (1974). The adsorption of Pseudomonas aeruginosa pilus-dependent bacteriophages to a host mutant with nonretractile pili. *Virology* 58**,** 149-163.

Bröms, J.E., Edqvist, P.J., Carlsson, K.E., Forsberg, Å., and Francis, M.S. (2005). Mapping of a YscY binding domain within the LcrH chaperone that is required for regulation of *Yersinia* type III secretion. *J Bacteriol* 187**,** 7738-7752.

Francis, M.S., Lloyd, S.A., and Wolf-Watz, H. (2001). The type III secretion chaperone LcrH co-operates with YopD to establish a negative, regulatory loop for control of Yop synthesis in Yersinia pseudotuberculosis. *Mol Microbiol* 42**,** 1075-1093.

Fürste, J.P., Pansegrau, W., Frank, R., Blöcker, H., Scholz, P., Bagdasarian, M., and Lanka, E. (1986). Molecular cloning of the plasmid RP4 primase region in a multi-host-range tacP expression vector. *Gene* 48**,** 119-131.

Hanahan, D. (1985). "Techniques for transformation of *E. coli*," in *DNA Cloning. A practical approach.,* ed. D.M. Glover. (Oxford, United Kingdom: IRL Press Ltd), 109-136.

Morales, V., Bäckman, A., and Bagdasarian, M. (1991). A series of wide-host-range low-copy-number vectors that allow direct screening for recombinants. *Gene* 97**,** 39-47.

Simon, R., Priefer, U., and Pühler, A. (1983). A broad host range mobilisation system for in vivo genetic engineering: transposon mutagenesis in Gram negative bacteria. *Nature Biotechnology* 1**,** 784-791.
